# Statistical analysis plan for Fluid Optimisation in Emergency Laparotomy (FLO-ELA) Trial; a multi-centre randomised trial of cardiac output-guided fluid therapy compared to usual care in patients undergoing major emergency gastrointestinal surgery

**DOI:** 10.1186/s13063-026-09552-3

**Published:** 2026-04-17

**Authors:** Neil J. Walker, Gordon Forbes, Brennan C. Kahan, Melanie Smuk, Rachel Phillips, Olivier Quintin, Kim May Lee, Mark R. Edwards

**Affiliations:** 1 Pragmatic Clinical Trials Unit, Wolfson Institute of Population Health, Queen Mary University of London, London, Queen UK; 2 Department of Biostatistics & Health Informatics, Institute of Psychiatry, King’s College London, Psychology & Neuroscience, London, UK; 3 MRC Clinical Trials Unit at UCL, University College London, London, UK; 4 Centre for Genomics & Child Health, Blizard Institute, Queen Mary University of London, London, UK; 5 Imperial Clinical Trials Unit, School of Public Health, Imperial College London, London, UK; 6 Department of Anaesthesia, Southampton General Hospital, University Hospital Southampton NHS Foundation Trust, Tremona Road, Southampton, SO16 6YD UK

**Keywords:** Statistical analysis plan, Days alive and out of hospital, Mortality, Length of stay, Emergency laparotomy, Perioperative care, Estimand

## Abstract

**Background:**

FLO-ELA is a multi-centre, parallel-group, open-label, randomised controlled trial (RCT) looking at the effect of minimally invasive cardiac output monitoring in the management of patients aged 50 years and above undergoing emergency gastrointestinal surgery. The primary outcome is days alive and out of hospital within 90 days of randomisation (DAOH-90). This article describes the statistical analysis plan (SAP) for this trial. The estimand and analytical approach presented here may be appropriate for clinical trials in a similar setting (critical care surgery).

**Methods and design:**

FLO-ELA is a superiority trial, incorporating an early-stage pilot phase, conducted at 63 UK hospitals. Participants are randomised on a 1:1 basis to the intervention or control group using a minimisation algorithm which balances on the following two factors (i) age, split into 3 groups, and (ii) American Society of Anesthesiologists (ASA) category, 5 categories on an ordinal scale reflecting participants’ baseline co-morbidities. In this SAP, we define the estimands and strategies for handling intercurrent events for each study outcome, then give a detailed description of planned analyses including general analytical principles, analytical framework for primary, secondary and process measure outcomes, approach to missing data and supplementary analyses looking at heterogeneity in treatment effect between key subgroups and potential differences in outcomes pre and post onset of Covid pandemic in the UK.

**Trial registration:**

ISRCTN 14729158. Registered on 02 May 2017.

**Supplementary Information:**

The online version contains supplementary material available at 10.1186/s13063-026-09552-3.

## Background, objectives and outcomes

Over 30,000 patients undergo major emergency abdominal surgery (laparotomy) each year in the UK. The potentially life-threatening nature of the underlying conditions necessitating this surgery, combined with the major physiological stress of surgery and patient co-morbidity, means that there is a significant healthcare burden of morbidity and mortality after this operation. Administration of intravenous fluids is a core activity during such major surgery. Protocolised approaches to the management of fluids and the cardiovascular system using advanced monitoring during surgery have been trialled as interventions. While they appear hopeful in reducing complications after major scheduled surgery on the gastrointestinal tract, there is insufficient evidence to support such interventions in the emergency surgery setting [1, 2].

### Setting

FLO-ELA is an open-label, multi-centre, randomised controlled trial (RCT) being undertaken at UK hospitals with emergency bowel surgery capacity either participating in the National Emergency Laparotomy Audit (NELA; England and Wales) or in a hospital in Scotland with equivalent emergency bowel surgery. Randomisation commenced on 8 September 2017 and ended with the attainment of the target of 3,138 participants; in fact the total recruited went above this to 3,140 the last of whom was recruited on 28 November 2024 [3]. The intervention being evaluated is cardiac output-guided haemodynamic therapy during, and for six hours after surgery, compared against current NHS treatment standard, i.e. intravenous fluid administration without the use of cardiac output monitoring. Patients must be aged 50 years or above to be eligible for inclusion. Further details on the rationale of the study, the nature of the intervention and inclusion/exclusion criteria can be found in the FLO-ELA protocol publication [3].

### Objective

The primary objective of the FLO-ELA trial is to establish whether surgery carried out under intervention conditions leads to better patient prognosis in terms of both the risk of post-surgery mortality and the number of days spent in hospital post-surgery. These measures are both captured in the primary outcome for this study, days alive and out of hospital within 90 days (DAOH-90). This is a superiority trial in which the alternative hypothesis (improved patient outcome in intervention compared to control group participants reflected in reduced mean DAOH-90) is tested against a null hypothesis in which no underlying difference in DAOH-90 is present between the two treatment arms. Results will provide evidence regarding the effectiveness and cost-effectiveness of cardiac-output guided haemodynamic therapy as a tool in the perioperative care of this population of patients.

### Statistical analysis plan

This article is a summary of the FLOELA SAP, version 5.0 (25th October 2025), this in turn aligns with the trial protocol, version 5.0 (14th November 2024), a summarised version of which is available in article format [3]. The full document is included as a supplementary file; note that the checklist [4] (also included as supplementary material) pertains to the SAP itself and not all the points are covered in this article.

### Outcomes

#### Primary outcome measure

The primary outcome (DAOH-90) is a quantitative measure capturing patients’ post-surgical experience and is a patient-centred outcome as defined by the Standardised Endpoints in Perioperative Medicine (StEP) initiative [5]. Following a previous example [6], this is to be calculated by subtracting the total number of days spent in hospital (including readmissions following discharge after the index surgery) from 90 for the subgroup of participants who survive 90 days or more post-randomisation, whereas for those who die within 90 days, the outcome is set to zero. Dates in relation to the index admission triggering recruitment into the study will be available from NELA data, whereas readmission and date of death will be obtained from Hospital Episode Statistic (HES) and Office for National Statistics (ONS) mortality data respectively, both of which are held and provided on request by NHS England [7, 8].

#### Secondary outcomes

Secondary outcomes are:

- Mortality within 90 days of randomisation (0 = alive at 90 days, 1 = died within 90 days post-randomisation).
- Mortality within 1 year of randomisation (0 = alive at 365 days, 1 = died within 365 days post-randomisation).

#### Process measures

Process measures are:

- Duration of hospital stay (number of days from randomisation until hospital discharge).
- Duration of stay in a level 2 or level 3 critical care bed within the primary hospital admission post-randomisation.
- Hospital readmission (and the time to readmission) as an inpatient (overnight stay) within 90 days from randomisation.

These outcomes reflect patient experience in the NHS hospital system. The first two of these are captured in the NELA database and in the case of duration of stay, this will be supplemented by mortality data from NHS England for the purposes of studying the competing risk in survival. The hospital readmission outcome is to be derived from HES data and supplemented with mortality data in a similar manner as for the above competing risk analysis.

## Sample size and calculation

The target sample size has evolved during the course of the study. The initial target based on the original binary primary outcome (mortality at 90-days post-randomisation) was *N* = 7,646. However, challenges to recruitment primarily in relation to the Covid-19 pandemic, and a lower than anticipated primary outcome event rate, necessitated a change in strategy. The original target was revised downward to a figure of 3,138 in tandem with a change in primary outcome to DAOH-90 whilst retaining 90% statistical power. Further details of and rationale underlying both the original and revised calculation are given in the appendix.

### Randomisation protocol

After enrolment but before the start of surgery, participants are allocated to treatment groups in a 1:1 ratio by minimisation with a random component (80% probability of allocation to treatment arm minimising imbalance). The minimisation factors are patient age (50–64 years, 65–79 years, and 80 + years) and American Society of Anesthesiologists (ASA) class as a measure of patient health pre-surgery; grades I, II, III, IV, and V [9]. Randomisation is performed as close as possible to the start of anaesthesia, typically when the patient arrives in the theatre suite for surgery. Further details of the randomisation process are provided in the protocol paper [3].

Whilst the trial is open-label with respect to participants and surgical staff, the trial statisticians and other key members of the trial team are blinded and will only receive patient treatment group information at the time of final analysis once the database has been locked.

### Interim analyses

The FLOELA trial’s data monitoring and ethics committee (DMEC) will review outcome data, safety data and recruitment data periodically during the trial and will recommend that the trial be stopped early if:

i. There is overwhelming evidence that is likely to convince a broad range of clinicians, including those supporting the trial and the general clinical community, that one trial arm is clearly indicated or contraindicated, and there was a reasonable expectation that this new evidence would materially influence patient management.
ii. It becomes evident no clear outcome will be obtained.

No formal stopping rules are in place and no adjustments to the primary analysis will be made to account for any interim analysis performed for the DMEC. All unblinded analyses for the DMEC will be performed by an independent statistician who is not otherwise involved in the trial.

### Participant flow

The flow and number of participants at different stages of the trial will be reported using a CONSORT [10] diagram (Appendix 1). This will show (i) the number of patients assessed for eligibility for inclusion in FLO-ELA (ii) the number excluded at the assessment stage for not meeting all inclusion criteria (iii) the number of participants randomised to each treatment arm (iv) the number of instances of non-adherence in the intervention arm and contamination in the control arm, and (v) the final number of participants included in the analysis of the primary outcome.

### Estimands for primary and secondary outcomes

Inference on the primary and both secondary outcomes is complicated by the potential occurrence of intercurrent events. Here we describe the estimand for the analysis of the primary and secondary outcomes, the intercurrent events anticipated in relation to FLO-ELA and how each intercurrent event will be handled in the estimand definition [11, 12].

#### Primary outcome

The estimand for the primary outcome is the difference in mean DAOH-90 between protocolised cardiac output-guided haemodynamic therapy and usual care, regardless of adherence or use of cardiac monitoring in the control arm, in patients aged ≥ 50 years who undergo emergency bowel surgery, are eligible for the study and are not lost to follow-up (Table 1).

**Table 1:** Estimand for analysis of primary outcome

Aspect	Definition
Target population	Patients ≥ 50 years old who undergo emergency bowel surgery
Variable/endpoint	Days alive and out of hospital within 90 days of randomisation (DAOH-90) = count of days alive and out of hospital within 90 days of randomisation where DAOH-90 = 0 if patient dies within 90 days and DAOH-90 = 90 − (days in hospital within 90 days of randomisation) if patient is alive 90 days after randomisation
Treatment conditions	Intervention Group—Protocolised cardiac output-guided haemodynamic therapy during surgery, and for six hours after surgery in patients admitted to a site capable of delivering this intervention Usual Care Group—Intravenous fluid administration without the use of cardiac output monitoring or protocol
Population level summary measure	Difference in means (Intervention v usual care group)
Intercurrent events	Strategy
Surgery not received (applies to both treatment arms)	Principal stratum (patients who undergo surgery regardless of treatment allocation)
Procedure modified after surgery begins such that no longer eligible for NELA (applies to both treatment arms)	Treatment policy
Receipt of cardiac output monitoring (control arm only)	Treatment policy
Failure to initiate cardiac output monitoring during/after surgery (intervention arm only)	Treatment policy
Cardiac output monitoring initiated but intervention algorithm not followed	Treatment policy

We will retain in analysis participants whose ultimate surgical procedure is discovered post-randomisation to have been ineligible for NELA (pending other inclusion/exclusion criteria), as these individuals fall within the target population as defined in Table 1. Exclusion of patients randomised in error will not lead to systematic bias in treatment effect estimates as the exclusions are based on pre-randomisation information which will not systematically differ between treatment arms.

Apart from the principal stratum strategy adopted in relation to patients undergoing/not undergoing surgery, all other intercurrent events listed above will be dealt with according to a treatment policy such that all patients receiving surgery will be included in analysis and deemed to belong to the group they were assigned to at randomisation.

#### Secondary outcomes

Secondary outcomes will be analysed according to the same overarching strategy with the difference that the summary measure will be the odds ratio in the outcome (mortality at 90 or 365 days) between the intervention and control groups.

## Analysis

### General analysis principles

All eligible, randomised participants who proceed to surgery and have a recorded outcome will be included in analysis conditional on additional criteria specified below, and will be considered in analysis to belong to the treatment group to which they were randomised. Patients who do not ultimately receive surgery will be excluded from analysis (see estimand framework section above). Patients with missing outcome data will be excluded from the analysis as will those who are found post-randomisation to have been ineligible on any criteria for inclusion in the study. A comprehensive list of inclusion/exclusion criteria is given in the FLO-ELA protocol paper [3].

Treatment effect estimates, associated 95% confidence intervals and two-sided *p*-values will be presented in relation to the analyses of the primary, secondary and all outcomes where a regression analysis is carried out. All inference will be based on two-sided 5% significance level. For each analysis, the number of individuals included in analysis will be reported.

### Baseline characteristics

Baseline characteristics will be summarized for each treatment group by the mean and standard deviation or median and interquartile range for continuous variables, and the number and percent for categorical variables.

### Estimator for treatment effect on DAOH90

The treatment effect in relation to the primary outcome (DAOH-90) will be estimated using a linear mixed model (LMM) with hospital site as a random effect. For this analysis, we will use the *mixed* procedure in Stata, which by default uses a maximum likelihood (ML) algorithm. The DAOH-90 outcome is anticipated to have a zero-inflated distribution; whilst this distribution is non-standard the use of a linear model has been supported elsewhere in the analysis of such data [13] and has the advantage of yielding a treatment effect which is readily interpretable. The adjusted mean difference (intervention vs control) from this model will be presented along with the 95% CI and *p*-value.

Earlier versions of the analysis plan described a negative binomial approach to analysis of the DAOH-90 data [3]. Simulation work in relation to another trial with a similar outcome suggested the negative binomial approach may perform poorly in terms of statistical power in relation to a simple regression approach, without an increase in Type I error (anecdotal, trial in early stages). Furthermore, the revised approach has the advantage of yielding an intuitive treatment effect and as indicated above is supported in the literature [13].

Participants for whom we cannot derive the primary outcome will be omitted from the analysis. This is an issue of missing data and falls outside the estimand strategy. The number of individuals excluded from the analysis for this reason will be reported as per the CONSORT template (Appendix 1).

We will not perform any model checks to evaluate assumptions such as normality of residuals or presence of outliers, as the planned analytical strategy will provide valid results even if these assumptions are violated. For instance, the primary outcome (DAOH90) comprises a bounded scale (this is also true for our process measure outcomes) and so will not have major outliers. Similarly, the planned analytical strategy is robust to deviations from the assumption of normality of residuals, and so will provide valid results even if this is not the case.

### Secondary outcomes

Both secondary outcomes (mortality within (i) 90 days and (ii) 1 year respectively) will be estimated according to the same principles laid out in the estimand framework in Table 1 except that the variable/endpoint in both cases is a binary indicator of mortality and the summary statistic is the odds ratio of mortality for the intervention group relative to control group.

### Covariate adjustment

The analysis for the primary outcome and for both secondary outcomes will be adjusted for the following covariates fitted as fixed effects: the minimisation factors of patient age and ASA class [14], urgency of surgery (Immediate, Urgent, and Expedited), Glasgow Coma Score (GCS), systolic blood pressure, and pulse rate [15], and a random intercept for the effect of hospital. Urgency of surgery and ASA class will be included as categorical variables, while patient age, GCS, systolic blood pressure, and pulse rate will be included as continuous variables. Patient age and GCS will be included assuming a linear association with the outcome, and systolic blood pressure and pulse rate will be included using restricted cubic splines with 3 knots (knots will be placed based on Harrell’s recommended percentiles: 10th percentile, 50th percentile and 90th percentile of covariate) [16, 17].

Missing data for baseline covariates to be included in the analysis of the primary outcome (and in all other analyses where covariates are included) will be accounted for using mean imputation for continuous variables, and a missing indicator variable for categorical variables [18].

A shell table for the presentation of the results of analysis of the primary and secondary outcomes is given in Appendix 2. In addition to results from these models, we will present Kaplan–Meier (KM) plots of the difference in survival profiles between participants in the two treatment groups with respect to the secondary outcomes.

### Additional analyses

#### Subgroup analyses

In order to assess potential heterogeneity of treatment effect across different patient groups, the analysis on the primary outcome will be repeated on subgroups of select categorical variables, namely (i) urgency of surgery (ii) indications for surgery (iii) gender (iv) pre or post Coronavirus disease 2019 (COVID-19) pandemic, i.e. whether randomised into study before or after 30th January 2020 (v) COVID-19 status for those whom these data are available from NELA records. In each case this will involve fitting the full model as specified for the main analysis plus the categorical variable in question plus its interaction with treatment group. We will also carry out subgroup analysis with respect to two continuous variables, age and NELA pre-operative risk score which in this case will be fit using cubic splines based on 3 knots according to recommendation [16, 17] and the associated interaction with treatment arm. All subgroup and spline interactions will be reported with 95% confidence intervals (CIs) and presented graphically for illustration. A significant subgroup effect will be inferred if the Wald test for the interaction between the subgroup variable and treatment group yields *p* < 0.05. In addition to the requirement for a recorded outcome, subgroup analysis will only include those patients who have recorded data for the subgroup variable in question.

#### Additional COVID-19 presentations

In addition to the COVID-19 analyses forming part of the subgroup analyses in relation to the primary outcome, we will also consider the effect of the COVID-19 pandemic on quality of treatment delivery and the pre-surgical risk profile of FLO-ELA participants. Treatment compliance rates (adherence and non-contamination as defined earlier) will be presented pre and post pandemic for all participants for whom this data is available (not separated by treatment group). Similarly, mean NELA mortality risk score and NELA standard of care measures will be presented on all available data pre and post pandemic.

#### Process measures analyses

We will include analyses of the following process measures capturing patients’ hospital experience; (i) duration of stay in a hospital bed post-randomisation (ii) time to readmission as a hospital inpatient (iii) duration of stay in a level 2 or 3 critical care bed post-randomisation within index admission. The first two of these will be analysed with a competing risk TTE model [19] using Stata’s stcrreg command to allow for the possibility of mortality within a 90 day timeframe and the outcome in (iii) will be analysed using a mixed-effects negative binomial regression. Summary statistics to accompany all of the above analysis results will be presented and KM plots presented with respect to analyses (i) and (ii).

A shell table for the presentation of process measure analysis results is given in Appendix 3.

#### Days at home analysis

The primary outcome may be considered a proxy for days *at home* within 90 days (DAH-90). However, we will not have sufficiently detailed data to track individual pathways in terms of residence outside of hospital for everyone in the database.

In order to assess if inference on DAOH-90 may be extended to DAH-90, we will analyse data for a subset of FLO-ELA patients for whom post-discharge destination (“home” or “residence other than own home”) is recorded. This was recorded as part of the NELA audit up to December 2019, but not thereafter. DAH-90 will be calculated in the same way as DAOH-90, except that in instances where a patient is discharged to residence other than own home, DAH-90 will be set to zero. The primary analysis on DAOH-90 will be repeated with DAH-90 for patients with available data. This will be compared against results of the primary analysis on DAOH-90 for (i) all patients (ii) the subset of patients on which DAH-90 analysis carried out for direct comparability.

#### Adherence and contamination

Adherence and contamination in the intervention and control group respectively are defined thus:

- Adherence (intervention group only): A cardiac output monitor used during surgery, and one or more cycles taken through the algorithm.
- Contamination (control group only): this is defined as a cardiac output monitor being used for a patient in the control group.

Rates of adherence and contamination respectively will be reported at end of study along with a detailed breakdown of reasons underlying instances of non-adherence and contamination.

#### Safety and protocol deviations

We will present (i) the number of serious adverse events (SAEs) and (ii) the number of patients experiencing a SAE by treatment group in addition to details on protocol deviations, by treatment group also.

All analyses additional to the analysis of the primary and secondary outcomes will be carried out using the same population as described (or subset thereof e.g. in the analysis of Covid-status which has only been recorded since 2020).

#### *Post hoc* analyses

Any analyses falling outside the scope of the SAP will be considered *post hoc* and reported as such.

### Timing of analysis

All analyses to be included in the final report will be carried out once all data have been collated from the various sources and the database has been locked.

### Software

All analyses described in this SAP (including interim analyses for the DMEC) are to be carried out in Stata version 19.0 [20].

## Discussion

This SAP elaborates on the summary of analyses of key outcomes in the already-published FLO-ELA protocol paper. In publishing ahead of database lock (and signing off the trial SAP of which this paper is an abridged version), we aim to demonstrate transparency with respect to the choice of analysis and study timelines and we recommend the publishing of SAPs in journals becomes common practice. We recommend and encourage the early development of an estimand in RCTs in order to establish full clarity in relation to the research question in the face of anticipated intercurrent events in the trial population.

The primary analysis is conducted assuming outcome data is missing at random, conditional on the covariates in the model. Bias may arise if this assumption is false, and outcome data is not missing at random, although it seems inconceivable that missingness in the primary outcome should vary systematically between the two treatment groups. Even if this were the case, we anticipate the rate of missing outcome data to be low since the outcome will be derived from routinely held data (HES and ONS mortality) and likely to be be available for the vast majority of study participants. A degree of missingness may also be present in model covariates, although preliminary data checks (e.g. from reports to the FLO-ELA DMEC) indicate that this will only affect a small number of participants and these individuals will not be lost from analysis since the missing data will be imputed in these instances.

Another key assumption underpinning the analysis of the primary outcome is that treatment assignment has no impact on whether patients undergo surgery or not (as the analysis population excludes patients who do not receive surgery in order to estimate a principal stratum strategy [21]). This assumption is justified based on contextual knowledge, i.e. in most cases, the relevant decision makers will be unaware of trial group allocation until surgery starts (i.e. at the point the decision is made). Further, the decision not to proceed with surgery has large health implications for the patient, and is only undertaken in response to a major change in the patient’s clinical condition since surgery was initially planned, and it is implausible that such a fundamental change in patient care would be undertaken on the basis of the planned method of fluid delivery. The analysis population will also exclude participants who were randomised in error (i.e. those who did not meet the eligibility criteria at the time of randomisation), as they fall outside the target population. However, this exclusion is based on pre-randomisation information and as such will be unbiased.

Whilst every trial is unique, we propose this set of estimands may be suitable for use in other trials conducted in critical care and surgery settings in which similar intercurrent events are likely to be encountered.

## Supplementary Information

Supplementary Material 1.

Supplementary Material 2.

## Data Availability

Not supplied, stand-alone article.

## Appendix 2

### Shell table; results for analysis of primary and secondary outcomes

**Table:**

	Number included in analysis		Summary measure			
	Intervention no. (%)	Usual care no. (%)	Intervention	Usual care	Treatment effect (mean difference/odds ratio (95% CI))	*p*-value
Days alive and out of hospital within 90 days						
Mortality within 90 days of randomisation						
Mortality within 1 year of randomisation						

## Appendix 3

### Shell table; results for analysis of process measures

**Table:**

	Number included in analysis		Summary measure			
	Intervention no. (%)	Usual care no. (%)	Intervention	Usual care	Treatment effect (95% CI)	*p*-value
Duration of hospital stay for* survivors – median (IQR)						
Survived to hospital discharge – no. (%)	n/a	n/a			n/a	n/a
Not discharged by end of trial – no. (%)	n/a	n/a			n/a	n/a
Died in hospital – no. (%)	n/a	n/a			n/a	n/a
Duration of stay in a level 2 or level 3 critical care bed within the primary hospital admission** – mean (SD)						
Time to hospital readmission as an inpatient (overnight stay) within 90 days from randomisation* – median (IQR)						
Readmitted to hospital within 90 days – no. (%)	n/a	n/a			n/a	n/a
Survived to 90 days with no readmission – no. (%)	n/a	n/a			n/a	n/a
Died prior to 90 days and prior to any readmission – no. (%)	n/a	n/a			n/a	n/a

*Treatment effect is a hazard ratio **Treatment effect is a rate ratio, which is based on the ratio of mean length of stay in level 2 or 3 critical care

## Appendix 4

### Sample size calculation

#### Original calculation

The original sample size calculation was based on mortality within 90 days of randomisation (a binary outcome; 0 = survived to 90 days, 1 = died within 90 days), and predicated on an event rate in the control arm of 19%. Contemporary NELA data showed that in patients aged 50 or over, mortality 90 days after emergency laparotomy was 19.5% in 2014 and 18.8% in 2015. Meta-analysis of randomised trials of perioperative goal-directed haemodynamic therapy (GDHT) suggested a relative risk reduction in mortality at longest follow-up of ~15% (RR 0.86 [95% CI 0.74–1.0]) (22). This is considered to constitute a clinically important effect, since if the intervention became routine practice for all patients undergoing emergency laparotomy annually it could save several hundred lives each year.

With a 5% alpha level, 90% power, and assuming a 2% dropout rate and a 19% 90-day mortality rate in the usual care arm, it was calculated that 3,823 patients were required in each arm (7,646 total) to detect a risk ratio of 0.85 (equivalent to an absolute decrease from 19% to 16.15%) for the initial primary outcome.

#### Revised calculation

It became apparent during the recruitment period that the initial target sample of 7,646 was (a) unattainable within the scheduled study period due primarily to major trial disruption from the Covid-19 pandemic (b) unlikely to achieve the stated power of 90% due to a lower than expected event rate. The switch to the DAOH-90 outcome was motivated by a need to recover statistical power in light of these issues.

### Step 1

#### Parameters for revised calculation

We proceeded from the following information:

- Overall FLO-ELA 90-day mortality rate = 12.0% (this figure was based on FLO-ELA data from a report to DMEC in November 2019).
- Mean stay in hospital in NELA population = 15.93 (standard deviation = 13.62) days. This figure was based on summary NELA data on duration of hospital stay provided on request to the FLO-ELA team, November 2020.
- Assumed relative risk (intervention v control) = 0.85. This mirrors the effect size used in the original sample size calculation.
- Assumed reduction in mean hospital stay of 2 days (intervention v control). This was deemed to be a realistic figure based on relevant literature [22–28].

These parameters give the following estimates for mortality and length of stay in the two treatment arms;

1. Control. 90-day mortality = 13.0%*, mean duration of stay = 15.93 (SD = 13.62**) days
2. Intervention. 90-day mortality = 11.05%*, mean duration of stay = 13.93 (SD = 13.62**) days

*The empirical rate of 12.0% derived from the FLO-ELA population. This may be considered a composite figure since the numbers in the two treatment groups in the FLO-ELA population are approximately 50:50. Assumed mortality rates in the two arms are adjusted accordingly observing 85% relative risk.

**Assumed the same in the two groups.

### Step 2

#### Approximation of distribution of DAOH-90 in each group

DAOH-90 was simulated using RStudio [29] as follows.

1. We sampled 10000 number of binary events for 90-day mortality from a binomial distribution using the group-specific mortality rate.
2. An event indicates death within 90 days; in this instance we set DAOH-90 to zero.
3. Otherwise, survival up to and beyond 90 days is assumed; in these instances duration of stay in hospital was simulated from a normal distribution using the group specific means (and common standard deviation of 13.62) laid out above. DAOH-90 was then calculated as 90 days – duration of stay.

This simulation yielded the following parameters for DAOH-90 in the respective treatment arms;

- Control; Mean = 64.45 days, S.D. = 27.96
- Intervention: Mean = 67.67 days, S.D. = 27.09

This equates to an expected mean difference of ~ 3.2 days between the treatment arms. The parameters underlying this calculation were chosen such that this is anticipated to be proportionate to the treatment effect included in the original sample size calculation and therefore of equivalent clinical significance.

### Step 3

#### Final sample size calculation

The above values were entered into a sample size calculation using Stata’s *power twomeans* function, which allows for specification of group specific variance. As in the original calculation, power was fixed at 90% and Type I error rate at 5%. This gives a sample size of 3,074 for a superiority trial. Correcting for anticipated 2% loss to follow up gives a revised sample size of 3,138 (rounded to nearest even number) to estimate a 3.2 day difference in DAOH-90.

## Abbreviations

ASA: American Society of Anesthesiologists
CI: Confidence interval
CONSORT: Consolidated Standards of Reporting Trials
COVID-19: Coronavirus disease 2019
DAH-90: Days at home within 90 days
DAOH-90: Days alive and out of hospital within 90 days
DMEC: Data monitoring and ethics committee
FLO-ELA: Fluid Optimisation in Emergency Laparotomy
GCS: Glasgow Coma Score
GDHT: Goal-directed haemodynamic therapy
HES: Hospital Episode Statistics
KM: Kaplan-Meier
LMM: Linear mixed model
ML: Maximum likelihood
NELA: National Emergency Laparotomy Audit
OR: Odds ratio
RCT: Randomised controlled trial
SAP: Statistical analysis plan
TTE: Time to event analysis
