# Supplementary material for: Statistical analysis plan for Fluid Optimisation in Emergency Laparotomy (FLO-ELA) Trial; a multi-centre randomised trial of cardiac output-guided fluid therapy compared to usual care in patients undergoing major emergency gastrointestinal surgery
Source: Trials. 2026 Apr 17;27:397. doi: 10.1186/s13063-026-09552-3 (PMC13217997; doi:10.1186/s13063-026-09552-3)
Supplement: Supplementary file 2 — Supplementary Material 2. [file 13063_2026_9552_MOESM2_ESM.pdf]

FLOELA

FLuid Optimisation in Emergency LAparotomy

Open, multicentre, randomised controlled trial of cardiac output-guided haemodynamic therapy compared to usual care in patients undergoing emergency bowel surgery.

Short Title *FLO-ELA trial*

Sponsor *University Hospital Southampton NHS Foundation NHS Trust*

The contact person for the above sponsor organisation is:
Dr Karen Underwood
R&D Senior Contracts Manager
R&D Department
E-level, SCBR Mail Point 138
Southampton General Hospital
Tremona Road
Southampton SO16 6YD
Email: karen.underwood@uhs.nhs.uk

IRAS Project ID *214459*

Chief Investigator *Dr Mark Edwards*

UK co-ordinating centre
Research Office
Department of Anaesthesia
Southampton General Hospital
Tremona Road
Southampton SO16 6YD
Email: admin@floela.org

Clinical Trials Unit
Pragmatic Clinical Trials Unit (PCTU)
Centre for Primary Care and Public Health
Blizard Institute
Yvonne Carter Building
58 Turner Street
London E1 2AB

Funder *National Institute for Health and Care Research Health Technology Assessment ref: 15/80/54*

FUNDED BY NIHR | National Institute for Health and Care Research

Contents

1 GLOSSARY OF TERMS AND ABBREVIATIONS 3

2 SIGNATURE PAGE 5

3 SUMMARY 6

4 INTRODUCTION 8

5 TRIAL OBJECTIVES 9

6 TRIAL METHODOLOGY 12

7 TRIAL PROCEDURES 15

8 STATISTICAL CONSIDERATIONS 28

9 RESEARCH ETHICS 32

10 DATA HANDLING AND RECORD KEEPING 33

11 PRODUCTS, DEVICES AND TECHNIQUES 34

12 SAFETY REPORTING 34

13 MONITORING & AUDITING 36

14 TRIAL MANAGEMENT & COMMITTEES 37

15 FINANCE AND FUNDING 38

16 SPONSORSHIP & INDEMNITY 38

17 PUBLICATION 38

18 REFERENCES 39

APPENDIX 1: NELA INCLUSION & EXCLUSION CRITERIA 43

APPENDIX 2: NELA DATA ENTRY 46

APPENDIX 3 - LEVEL OF CARE AFTER SURGERY 70

APPENDIX 4 – TYPICAL COMPLICATIONS OF EMERGENCY LAPAROTOMY 71

APPENDIX 5 – PROTOCOL VERSION HISTORY 72

1 GLOSSARY OF TERMS AND ABBREVIATIONS

AE	Adverse Event
ASA	American Society of Anesthesiologists
BP	Blood pressure
CEAC	Cost-Effectiveness Acceptability Curve
CHI	Community Health Index (Scotland)
CI	Chief Investigator
COVID-19	Coronavirus disease
(e)CRF	(electronic) Case Report Form
DAOH-90	Days Alive and Out of Hospital (within 90 days)
DMEC	Data Monitoring & Ethics Committee
EPOCH	Enhanced Peri-Operative Care for High-risk patients trial
EQ-5D-3L	Euro-Qol 5-Dimension 3-level quality of life measure
GCS	Glasgow Coma Scale
GDHT	Goal directed haemodynamic therapy
GCP	Good Clinical Practice
H&C Number	Health and Care Number (Northern Ireland)
HES	Hospital Episode Statistics
HR	Heart rate
HSCIC	Health & Social Care Information Centre (now NHS Digital)
ICU	Intensive Care Unit
NELA	National Emergency Laparotomy Audit
NHS	National Health Service
NHS REC	National Health Service Research Ethics Committee
NHS R&D	National Health Service Research & Development
NICE	National Institute for Health and Care Excellence
NSSISD	NHS National Services Scotland Information Services Division
ONS	Office of National Statistics
OR	Odds Ratio
Participant	An individual who takes part in a clinical trial
PCTU	Pragmatic Clinical Trials Unit
PI	Principal Investigator
PIS	Participant Information Sheet
(P)-POSSUM	(Portsmouth modified)-Physiological and Operative Severity Score for the enUmeration of Mortality and morbidity
QA	Quality Assurance

QALY	Quality Adjusted Life Years
QMUL	Queen Mary University London
RCT	Randomised Controlled Trial
REC	Research Ethics Committee
SAE	Serious Adverse Event
SOP	Standard Operating Procedure
TMG	Trial Management Group
TSC	Trial Steering Committee
UHS	University Hospital Southampton

2 SIGNATURE PAGE

Chief Investigator Agreement

The clinical study as detailed within this research protocol (version 5.0, 14/11/2024), or any subsequent amendments will be conducted in accordance with the Research Governance Framework for Health & Social Care (2005), the World Medical Association Declaration of Helsinki (1996) and the current and applicable regulatory requirements and any subsequent amendments of the appropriate regulations.

Chief Investigator Name: Dr Mark Edwards

Chief Investigator Affiliation: University Hospital Southampton NHS Foundation Trust

Signature and date 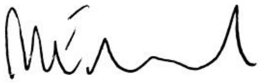 08/11/2024

Statistician Agreement

The clinical study as detailed within this research protocol (version 5.0, 14/11/2024), or any subsequent amendments will be conducted in accordance with the Research Governance Framework for Health & Social Care (2005), the World Medical Association Declaration of Helsinki (1996), Principles of ICH-GCP and the current and applicable regulatory requirements.

Statistician name: 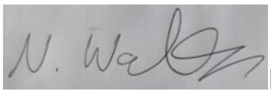 Neil Walker 11/11/2024

Principal Investigator Agreement

The clinical study as detailed within this research protocol (version 5.0, 14/11/2024), or any subsequent amendments will be conducted in accordance with the Research Governance Framework for Health & Social Care (2005), the World Medical Association Declaration of Helsinki (1996) and the current and applicable regulatory requirements and any subsequent amendments of the appropriate regulations.

Principal Investigator Name:

Principal Investigator Affiliation:

Signature and date:

3 SUMMARY

**Short title**	FLO-ELA trial
**Methodology**	Open, multi-centre, randomised controlled trial
**Research sites**	UK hospitals undertaking emergency bowel surgery. Hospitals in England and Wales must be participating in the National Emergency Laparotomy Audit (NELA)
**Objectives**	To establish whether minimally invasive cardiac output monitoring to guide protocolised administration of intra-venous fluid during and for up to six hours after major emergency bowel surgery will increase the number of days alive and out of hospital within 90 days of randomisation.
**Number of patients**	3138 patients (1569 per arm)
**Inclusion criteria**	Patients aged 50 years and over undergoing an expedited, urgent or emergency major abdominal procedure on the gastrointestinal tract eligible for inclusion within NELA.
**Exclusion criteria**	Refusal of patient consent, clinician refusal, abdominal procedure outside the scope of NELA, previous enrolment in the FLO-ELA trial, previous inclusion in the NELA audit within the same hospital admission, current participation in another clinical trial of a treatment with a similar biological mechanism.
**Statistical analysis**	Patients will be analysed according to the treatment group to which they were randomised and all eligible patients for whom an outcome is available and who proceeded to undergo surgery will be included in the analysis. Patients who were randomised in error (i.e. were ineligible at the time of randomisation) will be excluded from the analysis. Patients not proceeding to surgery post-randomisation will also be excluded.<br><br>Summary statistics, treatment effects, 95% confidence intervals, and p-values will be presented for primary, secondary and process outcomes. The primary outcome (days alive and out of hospital within 90 days of

	randomisation) will be analysed using a mixed-effects linear regression model with a random intercept for centre. The model will be adjusted for patient age, ASA score, urgency of surgery, and preoperative Glasgow coma score, systolic blood pressure, and heart rate. The secondary outcomes will be analysed using a mixed-effects logistic regression model with the same covariates and random-effect structure.
**Proposed start date**	01 May 2017
**Proposed end date**	31 January 2026
**Study duration**	105 months

4 INTRODUCTION

Emergency abdominal surgery on the gastrointestinal tract (laparotomy) is a common major surgical procedure performed for life-threatening abdominal conditions related to underlying cancer, infection or previous surgery. It is performed on over 30,000 patients in England and Wales each year (1,2) and has a particularly high burden of postoperative morbidity and mortality, with a 90-day postoperative mortality rate of 20% in those aged 50 and over. The critical need to improve the care of patients undergoing this procedure has been recognized by the establishment of a national audit of care and outcomes in this patient group (National Emergency Laparotomy Audit, NELA) and a number of national quality improvement initiatives (1,2). In a recent research priority setting exercise conducted by the Royal College of Anaesthetists and James Lind Alliance, research to improve outcomes for patients undergoing emergency surgery was chosen as one of the top ten priorities. This underlines the desire of clinicians, patients and the public to test treatments which may help improve outcomes for this group.

It is accepted that intra-venous fluids given during and after surgery have an important effect on patient outcomes, in particular following major gastrointestinal surgery. Yet they are commonly prescribed to subjective criteria leading to wide variation in clinical practice (3). One possible solution is the use of cardiac output monitoring to guide intra-venous fluid dosing as part of a haemodynamic therapy algorithm. This approach has been studied for many years and has been shown to modify inflammatory pathways, and improve tissue perfusion and oxygenation (4,5). A Cochrane review of this intervention was recently updated, incorporating the largest contemporary trial in this area to date (6). Complications were less frequent among patients treated according to a hemodynamic therapy algorithm (Intervention 488/1548 [31.5%] vs Controls 614/1476 [41.6%]; RR 0·77 [0·71-0·83]). The intervention was associated with a reduced incidence of post-operative infection (Intervention 182/836 patients [21·8%] vs Controls 201/790 patients [25.4%]; RR 0·81 [0·69-0.95]) and a reduced duration of hospital stay (mean reduction 0.79 days [0·62-0.96]). There was a non-significant reduction in mortality at longest follow-up (Intervention 267/3215 deaths [8.3%] vs Controls 327/3160 deaths [10.3%]; RR 0·86 [0·74-1·00]).

Despite this suggestion of benefit in elective surgery, these findings are not generalisable to patients undergoing emergency abdominal surgery (7). Patients requiring emergency bowel surgery have fundamental pathophysiological differences from elective patients. These include acute inflammation, sepsis, bleeding, and fluid disturbances which may be established before surgery even begins. They therefore have similarities with critically ill patients, in whom the evidence base for fluid resuscitation based on cardiac output monitoring is very uncertain (8–10). There is a lack of dedicated studies of this treatment in emergency surgical patients, with only one pilot study of a cardiac output-guided haemodynamic therapy algorithm exclusively in patients undergoing emergency laparotomy (11). Other studies included too few patients undergoing emergency surgery to allow subgroup analyses (12–15).

These data highlight the uncertainty surrounding the possible benefits of peri-operative haemodynamic therapy algorithms in emergency bowel surgery and the need for a definitive large multi-centre clinical trial to resolve this. The aim of this trial is to evaluate the effects of peri-operative haemodynamic therapy guided by cardiac output on the number of days spent alive and out of hospital by patients following major emergency bowel surgery. NELA, commissioned by the Healthcare Quality Improvement Partnership (HQIP) as part of the National Clinical Audit Programme in England and Wales, provides a detailed ongoing dataset and engaged clinical community in this patient group. This supports an efficient trial design with minimal supplementary data collection beyond that already collected routinely for NELA and national databases.

Protocol version 3.0 has introduced a modified primary outcome and sample size (see: sections 5.8 and 6.4.3). The change from the originally planned primary outcome (mortality at 90-days after randomisation) was made in response to lower-than-anticipated recruitment rates, due to COVID-19 and other issues, and a lower-than-predicted estimated control group mortality rate. The changes were made with the approval of the trial steering committee and trial funder. No members of the trial team, trial steering committee or funding body had access to, or knowledge of, ongoing trial results at the time the decision to modify the primary outcome was made.

Composite outcomes of mortality and time spent in hospital are efficient, patient-centred postoperative outcome measures recommended in perioperative core outcome sets (16,17). Days Alive and Out of Hospital within 90 days of randomisation was selected as an outcome measure that is statistically efficient, is expected to be modifiable by this intervention, is obtainable using data already being collected for the trial, and is of clear importance to patients and healthcare systems.

5 TRIAL OBJECTIVES

5.1 Primary objective

To establish whether the use of minimally invasive cardiac output monitoring to guide protocolised administration of intra-venous fluid (goal-directed haemodynamic therapy, GDHT), for patients aged 50 and over undergoing emergency laparotomy will increase the number of days spent alive and out of hospital within 90 days of randomisation, when compared with usual care.

5.2 Primary outcome measure

Days Alive and Out of Hospital within 90 days of randomisation (DAOH-90)

5.3 Secondary objectives

To determine whether GDHT reduces mortality 90 days and one year after randomisation, and is cost-effective.

5.4 Secondary outcome measures

- Mortality within 90 days of randomisation
- Mortality within one year of randomisation

5.5 Process measures

- Duration of hospital stay (number of days from randomisation until hospital discharge)
- Duration of stay in a level 2 or level 3 critical care bed within the primary hospital admission
- Hospital readmission as an inpatient (overnight stay) within 90 days from randomisation

5.6 Health economic endpoints

- Mean cost of the intervention and control treatments
- Mean cost of secondary care resource use within 90 days from randomisation
- Mean cost of secondary care resource use within one year from randomisation
- QALY gain at 90 days from randomisation using EQ-5D-3L-derived utility scores at baseline and 90 day follow-up (estimated from preceding EPOCH trial data)
- QALY gain at one year from randomisation using EQ-5D-3L-derived utility scores at baseline and one year follow-up (estimated from preceding EPOCH trial data)

5.7 Assessment of primary and secondary outcomes

DAOH is a validated postoperative outcome measure calculated as a composite of postoperative mortality (by assigning a value of zero days for any death within 90 days), length of index hospital stay post-randomisation, and the duration of any hospital readmissions (18). Using the 90-day timeframe after randomisation and as described previously (18), DAOH-90 will be calculated as follows:

- Participants who die within the 90 days following randomisation will be allocated a value of zero days
- For participants surviving to 90 days: DAOH-90 = 90 – (number of days spent in hospital within 90 days of randomisation).

The number of days in hospital is defined as an inpatient (overnight) stay in any hospital. It is made up of the initial postoperative stay in hospital for surgery (the number of days from randomisation until the patient is discharged) as well as any hospital readmissions (number of days spent in any hospital after discharge) up to day 90 after randomisation.

We will request hospital episode statistics and mortality data from NHS Digital for participants in England or equivalents for the devolved nations (NHS National Services Scotland Information Services Division, NSSISD, individual Healthcare Trusts (or the Business Services Organisation) in Northern Ireland and Patient Episode Database for Wales, PEDW). Prospective consent for Office of National Statistics (ONS), Hospital Episode Statistics (HES) and devolved nation equivalent data linkage will be sought before enrolment into the trial. Mortality outcomes will be derived from ONS data (for England and Wales; via NSSISD for Scotland and individual Healthcare Trusts in Northern Ireland). Duration of critical care stay during the index hospital admission will be derived from NELA data (or from a mirror electronic Case Report Form (eCRF) database in Scotland and Northern Ireland – see section 7.7). Duration of hospital stay during the index hospital admission, and during any hospital readmissions will be derived from HES, NSSISD, NI Heathcare Trusts and PEDW data.

5.8 Changes to planned outcomes

The originally planned primary outcome was mortality within 90 days of randomisation, requiring a sample size of 7646 participants. Following a review by the Trial Steering Committee (TSC) and trial funder in 2020-2021, the primary outcome was modified to DAOH-90 for protocol version 3.0 while recruitment was ongoing, with a consequent reduction in required sample size to 3138 (see sections 6.4, 8.1 and 8.2). The original primary outcome, 90-day mortality, is now included as a secondary outcome.

5.9 Estimand framework

Inference on the primary and both secondary outcomes is complicated by the potential occurrence of inter-current events. We illustrate how this will be dealt with using an estimand framework (see Table 1). The estimand for the primary outcome (DAOH90) is the difference in means of days alive and out of hospital within 90 days of randomisation between protocolised cardiac output-guided haemodynamic therapy vs. usual care (intravenous fluid administered without use of cardiac output monitoring), regardless of adherence or use of cardiac monitoring in the control arm, in participants aged ≥50 years who undergo emergency bowel surgery. The estimand for both secondary outcomes will be the odds ratio of mortality (at 90 days or 365 days) in the intervention relative to usual care arm, both of which as described above. Details on the analytical strategy underlying this approach are presented in Table 1.

Table 1 – Estimand framework for primary outcome analysis

Aspect	Definition
**Target population**:	Patients ≥50 years old who undergo emergency bowel surgery
**Variable/endpoint**:	Days Alive and Out of Hospital within 90 Days of Randomisation (DAOH90 = count of days alive and out of hospital within 90 days of randomisation where DAOH90 = 0 if patient dies within 90 days and DAOH = 90 – (days in hospital within 90 days of randomisation) if patient alive 90 days after randomisation)
**Treatment conditions:**	**Intervention Group -** Protocolised cardiac output-guided haemodynamic therapy during surgery, and for six hours after in patients admitted to an area capable of delivering this intervention.<br><br>**Usual Care Group -** Intravenous fluid administration without the use of cardiac output monitoring or protocol.
**Population level summary measure**	Difference in means (Intervention v usual care group).
**Intercurrent events**	**Strategy**
Surgery not received (applies to both treatment arms)	Principal stratum (of participants undergoing surgery)
Procedure modified after surgery begins such that no longer eligible for NELA (applies to both treatment arms)	Treatment policy
Receipt of cardiac output monitoring (control arm only)	Treatment policy
Failure to initiate cardiac output monitoring during/after surgery (intervention arm only)	Treatment policy
Cardiac output monitoring initiated but intervention algorithm not followed	Treatment policy

6 TRIAL METHODOLOGY

6.1 Study design

Open, multi-centre pragmatic randomised controlled trial with internal pilot.

6.2 Inclusion criteria

- Age 50 years and over
- Scheduled to undergo a surgical procedure which fulfils the criteria for entry into the National Emergency Laparotomy Audit (NELA), i.e. an expedited, urgent or emergency abdominal procedure on the gastrointestinal tract within the audit scope, as listed in Appendix 1
- Patient has an NHS/Community Health Index (CHI)/Health and Care (H&C) number

The term “emergency” laparotomy is defined in line with NELA and the National Confidential Enquiry into Peri-Operative Deaths (NCEPOD) 2004, to encompass the following categories: “immediate” surgery (required within two hours of the decision to operate), “urgent” surgery (required within 2-18 hours of the decision to operate) and “expedited” surgery (required within days of the decision to operate).

6.3 Exclusion criteria

- refusal of patient consent
- clinician refusal
- previous enrolment in the FLO-ELA trial
- previous inclusion in NELA within the current hospital admission
- current participation in another clinical trial of a treatment with a similar biological mechanism
- scheduled abdominal procedure outside the scope of NELA, as listed in Appendix 1.

A full list of NELA inclusion / exclusion criteria is included in Appendix 1. During the course of the trial the NELA Project Team may make minor modifications to the definitions of surgical cases included within the audit. In this circumstance the inclusion/exclusion criteria for FLO-ELA will be amended to ensure consistency with NELA. Although hospitals in Scotland/Northern Ireland (NI) are not participating in the NELA program, the same procedural eligibility criteria should be used.

6.4 Internal pilot

The FLO-ELA trial will incorporate an internal pilot in order to confirm predicted site enrolment, participant recruitment, representativeness of the participants recruited and compliance with the study protocol. The duration of the internal pilot will be the first 12 months of recruitment. During this time, it is anticipated that 100 sites will be activated, and approximately 1780 patients will have been randomised. Recruitment to FLO-ELA will continue during the internal pilot analysis. A report will be compiled at the end of the internal pilot phase, which will be discussed at a monitoring meeting with the funder.

6.4.1 *Internal pilot outcomes:*

- Number of sites open and having recruited first patient
- Number of patients randomised. This is anticipated to be approximately 1780 after 12 months. This figure allows for slower recruitment in the initial six months for each hospital (run-in phase).
- Adherence (intervention group): this is defined as a cardiac output monitor being used, and one or more cycles taken through the algorithm.
- Contamination (control group): this is defined as a cardiac output monitor being used for a patient in the control group.
- Representativeness of randomised patients compared with all eligible patients in the NELA dataset
  - age
  - sex
  - pre-operative physiological markers.
- Control arm event rate: the Data Monitoring and Ethics Committee will assess the 90-day mortality rate in the control arm to assess whether figures used in the sample size calculation are realistic. Only patients recruited during the first five months of recruitment will be included in this analysis; this is to provide enough time to complete data linkage. The trial team will remain blinded to this event rate.

6.4.2 *Internal pilot stop/go criteria:*

Number of sites open and having recruited at least one patient

- *>90 sites open and having recruited first patient*: continue.
- *70-90 sites open and having recruited first patient*: review site selection and initiation procedures, provide further support.
- *<70 sites open and having recruited first patient*: discuss urgently with Trial Steering Committee and funder, considering all options including discontinuation.

Number of patients randomised (target 1780)

- *>80% of recruitment target achieved (>1426 patients)*: continue.
- *50-80% of recruitment target achieved (890-1426 patients)*: consider recruitment strategies (opening more centres, further training and support).
- *<50% of recruitment target achieved (<890 patients)*: discuss urgently with TSC and funder, considering all options including discontinuation.

Adherence (intervention group)

- *>90%*: continue.
- *80-90%*: consider options such as re-training staff, providing further support, closing problem sites.

- *<80%*: discuss urgently with TSC and funder, considering all options including discontinuation.

Contamination (control group)

- *<10%*: continue.
- *10-20%*: consider options such as re-training staff, providing further support. Individual sites with contamination rates over 10% may be closed at the end of the pilot period.
- *>20%*: discuss urgently with TSC and funder, considering all options including discontinuation.

Representativeness of randomised patients compared against all eligible patients in the NELA dataset

- *Small differences in all variables (<5 years difference in age, <10% difference in gender, <10% difference in pre-operative mortality risk score)*: continue.
- *Large difference in one or more variables*: consider strategies to target specific groups.

A face-to-face workshop with hospital Principal Investigators will be held 15 months after recruitment begins to review contamination and adherence data and share best practice recruitment strategies.

6.4.3 Results from internal pilot:

Overall site and participant recruitment during the pilot phase were below target and were therefore reviewed by the TSC and funder. A revised recruitment trajectory was proposed and trial progress monitored. Due to the impact of COVID-19, recruitment was paused on 23 March 2020 and restarted on 01 September 2020. At the funder's request, a trial recovery proposal was submitted to address the effects of lower than anticipated recruitment rates, a lower than anticipated pooled mortality rate in the trial (see section 8.1), and the impact of COVID-19. The recovery proposal involved a change of the planned primary outcome measure with consequent reduction in required sample size, and an extended trial recruitment phase.

7 TRIAL PROCEDURES

7.1 Recruitment and screening

Potential participants will be screened by clinical and research staff at the site having been identified from operating theatre lists and by communication with the relevant nursing and medical staff. Due to the randomisation at an individual level, participant consent will be sought. Prior data suggests a majority of

eligible patients will have capacity to consent (19,20). However, this trial also seeks to include participants who are incapable of giving consent to enter the trial for a number of reasons:

1. Patients may be experiencing severe abdominal pain or vomiting and have received strong analgesia, or may require multiple medical interventions in the time available before surgery.
2. Patients with potentially life-threatening acute conditions may require surgery in an urgent time frame. During this limited time, priority must be given to medical information and consent for surgery.
3. Patients may lack mental capacity due to acute delirium, or sedation in an intensive care setting.

Furthermore, due to the unanticipated nature of this surgery, there would not be an opportunity to perform consent before admission to hospital. These patients are an established exception to the general rule of informed consent in clinical trials, in accordance with the Declaration of Helsinki 2013, the Mental Capacity Act 2005, and the Adults with Incapacity (Scotland) Act 2000 because:

- *The research is related to the impairing condition that causes the lack of capacity or to the treatment of those with that condition*; this is critical illness caused by an underlying condition needing urgent surgery.
- *The research cannot be undertaken as effectively with people who have the capacity to consent to participate*. Patients lacking capacity due to illness severity may be a subgroup with more to gain from optimal fluid management; excluding this subgroup would limit the representativeness of the overall FLO-ELA group and reduce the generalisability of the study findings to the ultimate target clinical group.
- *The research will serve to increase knowledge of the cause, treatment or care of people with the same or similar condition and that the risks to participants will be negligible, with no significant interference with their privacy or freedom of action.* We are testing the hypothesis that GDHT reduces mortality after surgery, demonstrating whether this intervention is beneficial to people with the same or similar conditions. The preceding literature suggests that the risk-benefit ratio is favourable (6). There will be no interference with privacy or freedom of action.

Having identified eligible participants at sites, research team members will assess whether the patient is capable of giving consent to trial participation.

7.2 Informed consent

7.2.1 Consent by patients

In patients with capacity, an authorised member of the team (named on the Delegation Log and with GCP training) will be responsible for obtaining written informed consent. This process will include an explanation of the aims, methods, anticipated benefits and potential hazards of the trial and provision of a Patient Information Sheet accompanied by the relevant consent form. The Principal Investigator or designee will

explain to all potential participants that they are free to refuse to enter the trial or to withdraw at any time during the trial, for any reason. Patients will be given an adequate amount of time to consider their participation in the trial. Within the time available before the patient proceeds to surgery the patient will be allowed to specify the time they wish to spend deliberating, and have a second consultation if they wish to consider and discuss again. Periods shorter than 24 hours to consider the trial will be necessary due to the emergency nature of the surgery, however the person seeking consent must be satisfied that the patient has fully retained, understood and deliberated on the information given. Patients who are not entered into this trial should be recorded (including reason not entered) on the patient-screening log in the FLO-ELA Investigator Site File.

7.2.2 Consultation for patients lacking capacity to consent (England, Wales and Northern Ireland)

In cases where the patient lacks capacity to give informed consent and a Personal Consultee is available to advise on the presumed wishes of the patient, and there is adequate time to undergo consultation, authorised staff will explain the FLO-ELA trial and provide a Consultee Patient Information Sheet. After checking that his has been understood, if the Personal Consultee agrees that the patient would want to participate, they will be asked to sign a Consultee Declaration Form. If the Personal Consultee is not present, agreement can be obtained by telephone, and a Consultee Telephone Agreement Form will be completed. If no Personal Consultee is available, a Nominated Consultee may be approached, agreement being addressed in the same manner as for the Personal Consultee.

A Personal Consultee is defined as someone who knows the person who lacks capacity in a personal capacity who is able to advise the researcher about the person who lacks capacity's wishes and feelings in relation to the project and whether they should join the research. A Nominated Consultee is defined as someone who is appointed by the researcher to advise the researcher about the person who lacks capacity's wishes and feelings in relation to the project and whether they should join the research. This may include a member of the care team or GP, as long as they have no connection with the research project.

7.2.3 Emergency consent for patients lacking capacity to consent (England, Wales and Northern Ireland)

Due to the emergency nature of the surgery, and the need to proceed with medical intervention – including fluid management – there may not be a Personal or Nominated Consultee available in a timely fashion. In other cases, a Personal Consultee may be available but the urgency of the surgery means there is inadequate time for the Consultee to receive trial information and to advise on the enrolment of the person who lacks capacity, particularly as clinical information must take priority. In these cases the authorised research team member will proceed with emergency consent using the process described in Section 32(9) of the Mental Capacity Act 2005. An independent doctor nominated by the local research team will be consulted - either in person or via telephone - and if they agree, the researcher will recruit the patient into

the trial. An Emergency Consent form will be completed by the member of the research team seeking consent.

7.2.4 Retrospective consent (England, Wales and Northern Ireland)

If a patient subsequently recovers capacity to consent, retrospective consent will be sought. This process will use the same approach as with a first approach to patients with capacity. However, as the intervention period occurs while the patient is under anaesthesia and for only up to six hours after surgery, in almost all cases the study intervention will be completed before the patient regains capacity. In these cases consent will allow data use, but no other contact with the patient for trial interventions will be required. Patients will not be informed of their treatment group allocation until after retrospective consent is obtained. Refusal of consent at this stage should be treated as a patient withdrawal from the study, see section: 7.10. Specific Retrospective Patient Information Sheets and Retrospective Consent Forms will be used. If however a site becomes aware that the patient has a pre-existing condition which means they would never regain sufficient capacity to give informed retrospective consent, agreement of a Personal or Nominated Consultee should be sought to use the patient's data.

7.2.5 Recruitment of Patients lacking capacity in Scotland

The Adults with Incapacity (Scotland) Act 2000 allows recruitment of participants incapable of giving consent in a similar manner to that described under the MCA 2005 in England and Wales (see: Section 7.2.3). Where there is adequate time before surgery, consent should be obtained from any guardian or welfare attorney who has the power to consent to the patient's participation in research or, where there is no such guardian or welfare attorney, from the patient's nearest relative. Discussion of trial participation may take place in person or via telephone.

In more urgent settings with limited time available, there may not be adequate time for a guardian, welfare attorney or nearest relative to consider the trial, particularly as clinical information must take precedence. In this setting in Scotland there is currently no provision for recruitment into this type of research in the emergency settings. Therefore these patients may not be recruited to the trial.

If a patient subsequently recovers capacity to consent, retrospective consent will be sought as described in section 7.2.4. If it becomes evident that the patient would never regain capacity, consent to use the patient's data will be sought from a guardian, welfare attorney or nearest relative.

7.3 Randomisation

After enrolment but before the start of surgery, participants will be centrally allocated to treatment groups in a 1:1 ratio by minimisation with a random component. The minimisation factors will be patient age (50-

64 years, 65-79 years, and 80+ years) and ASA class (I, II, III, IV, and V). Randomisation will be performed as close as possible to the start of anaesthesia, typically when the patient arrives in the theatre suite for surgery. To enter a patient into the FLO-ELA trial, research staff at the site will log on to a secure web-based randomisation platform provided by PCTU Queen Mary University of London and enter the patient's details to obtain a unique patient identification number and allocation to a treatment group. Allocation concealment will be used, ensuring that no one involved in study will be aware of the treatment allocation until after the patient has been randomised.

7.4 Trial treatment

The trial treatment period will commence at the start of general anaesthesia and continue for **six hours after the completion of surgery**. Eligible patients will be randomised to receive either cardiac-output guided haemodynamic therapy (intervention group), or usual care. Perioperative management for ***all*** patients during the trial treatment period will be in accordance with recommended guidance below.

7.4.1 Perioperative management for all patients

Care for all patients has been loosely defined to avoid extremes of clinical practice but also practice misalignment (21). All patients will receive standard measures to maintain oxygenation (SpO_2 ≥94%), haemoglobin (>80 g/L), and core temperature (36.5-37.5°C). A list of recommended fluids that may be given will be provided in the Standard Operating Procedure (SOP) for the study treatment. These fluids have a composition recommended by NICE for their specific clinical indication, i.e. maintenance fluid requirements or plasma volume expansion (22). A recommended maintenance fluid will be administered at 1ml/kg/hr. Mean arterial pressure will be maintained between 60 and 100 mmHg using a vasopressor or vasodilator as required. If inotropes, vasoconstrictors or vasodilators are required, they should be provided by intravenous infusion rather than intermittent bolus. Other aspects of perioperative care should be based on the best available evidence for this group (23,24), and the audit standards recommended by NELA (2).

7.4.2 Intervention group

The cardiac output-guided haemodynamic therapy intervention will commence with induction of anaesthesia and continue at least until the end of surgery. In patients receiving level 2/3 critical care after surgery, the intervention will continue for six hours after the end of surgery. This level of care may be delivered in intensive care units, high dependency units or post-anaesthetic care units (PACU). For patients with a clinical plan to be transferred to level 1 (ward) care after initial recovery from anaesthesia in the PACU, wherever possible the intervention should be delivered for six hours within the PACU before transfer. See Appendix 3 for definitions of levels of care. Cardiac output and stroke volume will be measured by cardiac output monitor. Clinicians may choose from a range of cardiac output monitors in established use which have been shown to track changes in cardiac stroke volume accurately. Please see

the SOP for the study treatment for a recommended list. No more than 500ml of intra-venous fluid will be administered within the intervention period prior to commencing cardiac output monitoring. In addition to the maintenance fluid described previously, patients will receive a 250ml fluid challenge with a recommended fluid (see: SOP for the study treatment), administered over five minutes or less. This fluid challenge will be repeated if there is evidence of fluid responsiveness, defined as ≥10% increase in stroke volume in response to the previous fluid challenge AND stroke volume variation (SVV) >5%. This will continue until a maximal value of stroke volume is achieved, defined as a stroke volume maintained for at least 20 minutes with no evidence of fluid responsiveness (see: SOP for the study treatment). Following major changes in haemodynamic status, such as following emergence from anaesthesia, further 250ml fluid challenge is recommended to re-establish the presence or absence of fluid responsiveness, and the maximal value of stroke volume revised if necessary. All other management decisions will be taken by clinical staff. If there is a clear clinical indication, the treating clinician may adjust both the volume and type of fluid administered, for example if there is concern about persistent hypovolaemia or fluid overload. Such decisions may relate to clinical circumstances or physiological measurements (e.g. pulse rate, arterial pressure, urine output, serum lactate, base excess).

7.4.3 Usual care group

Patients in the control group will be managed by clinical staff according to usual practice, without the use of cardiac output monitoring. In addition to the maintenance fluid, 250ml fluid challenges with a recommended intra-venous fluid will be given for plasma volume expansion. (see SOP for the study treatment) These will be administered at the discretion of the clinician guided by pulse rate, arterial pressure, urine output, core-peripheral temperature gradient, serum lactate and base excess. If a specific haemodynamic end-point for fluid challenges is to be used, the most appropriate would usually be a sustained rise in central venous pressure of at least 2 mmHg for 20 minutes or more. Patients should not be randomised if the clinician intends to use cardiac output monitoring regardless of study group allocation; this is considered ‘clinician refusal’ and is a specific exclusion criteria. However, clinical staff are able to request cardiac output monitoring if this is required to inform the treatment of a patient who becomes critically ill (e.g. because of severe haemorrhage); in this situation a protocol deviation form will be completed.

7.5 *Intervention algorithm*

General haemodynamic measures (all patients)

1. **Maintenance fluid (see SOP) at 1 ml/kg/hr**
2. **Transfuse blood to maintain haemoglobin >80 g/l**
3. **Clinician retains discretion to adjust therapy if concerned about risks of hypovolaemia or fluid overload**
4. **Mean arterial pressure 60-100 mmHg; $Sp0_2$ ≥94%; temperature 36.5-37.5°C; heart rate <100 bpm**

Administering fluid to a stroke volume end-point (intervention group)

1. **250ml fluid boluses to achieve a maximal value of stroke volume**
2. **Fluid challenges should not be continued in patients who are not fluid responsive in terms of a stroke volume increase**
3. **Fluid responsiveness is defined as a stroke volume increase ≥10% AND stroke volume variation >5%**
4. **If stroke volume decreases further fluid challenge(s) are indicated**
5. **Persistent stroke volume responsiveness suggests continued fluid loss**

What if blood or IV fluid is required regardless of stroke volume?

1. **If blood products or additional fluid challenges are required, then stroke volume should still be monitored to identify any change in maximal stroke volume**

7.6 *Blinding and procedures to minimise bias*

FLO-ELA is a pragmatic effectiveness trial of a treatment algorithm. It is not possible to conceal treatment allocation from all staff in trials of this type. Therefore, this trial will be open-label, and patients and the staff delivering the intervention will be unblinded. However, procedures will be put in place to minimise the possibility of bias arising because research staff become aware of trial group allocation. Clinicians will be instructed that the decision to admit a patient to critical care after surgery should be made on conventional clinical grounds before randomisation. Any changes to the planned level of care after surgery made after randomisation should also be based on clinical grounds and not trial group allocation. Confirmation of the primary and secondary outcomes is objective and automated through use of national registry data. While hospital discharge date may be influenced by potentially unblinded clinicians, the risk of bias is low because:

- separate teams are involved in delivering the intervention (anaesthesia/critical care) and overseeing later postoperative recovery and discharge (surgeons); the latter will typically be unaware of group allocation.
- given the long length of stay in hospital after emergency laparotomy, discharge decisions are made on average 10-14 days after the trial intervention has taken place.
- those involved in delivering the trial intervention will be instructed not to discuss or reveal group allocation to other members of the clinical care team.

Adjudication of Serious Adverse Events (SAEs) will be by the local Principal Investigator, who will be blinded to study group allocation.

Staff in hospitals participating in NELA are currently able to download a pseudo-anonymised NELA dataset for patients from their hospital that have been entered into NELA and have completed records. In order to prevent unauthorised local analysis, for hospitals participating in FLO-ELA, this dataset download will not indicate whether individual patients were recruited into FLO-ELA, and the data fields relating to the management of haemodynamic therapy, and the supplementary data fields used within FLO-ELA will not be included.

Research staff enrolling patients will not necessarily be blinded to previous allocations but the randomisation method used is not predictable so there is little risk of selection bias (25). The trial management group and the trial steering committee will not see results broken down by treatment arm during the trial. Final analysis will occur once all follow up data is collected, the final statistical analysis plan has been signed off and data cleaning has occurred. The trial statisticians and health economists will not have access to unblinded trial data (i.e. data with treatment allocation included, or variables which could predict treatment allocation such as compliance) until after the final statistical analysis plan and health economics analysis plan have been signed off and the database is locked for final analysis. The independent data monitoring committee will see outcome results by treatment group but the report will be

prepared by an independent statistician, not otherwise involved in the trial.

7.7 Data collection

In hospitals in England and Wales, nearly all data described below are already collected for NELA as part of routine care, under section 251 of the NHS Act 2006. NELA data are entered on to the secure online web portal locally by the range of clinicians caring for laparotomy patients, with each specialty entering data on their area of clinical care. Existing NELA leads at each hospital monitor data completeness, addressing any missing data and taking responsibility for completing and locking patient records. Data completeness is monitored routinely by the central NELA team and fed back to sites regularly as an audit standard. A small number of data fields will be added to the NELA web portal for FLO-ELA, only becoming activated for those patients who have given consent (or alternatives for those lacking capacity) and been randomised. Clinicians will be asked to complete these data fields prospectively, as they currently do for NELA. Research nurses will check for data completeness and accuracy of the FLO-ELA specific data fields after the intervention period. This will be monitored and actively managed throughout the trial. As NELA is not commissioned in Scotland and Northern Ireland, an eCRF database will be produced with identical NELA data fields to those used in FLO-ELA. Data entry to a secure online portal will be carried out by local research teams. This database will issue an eCRF ID in the same format to the NELA ID. Local investigators in all nations will also enter identifiable data onto the secure online randomisation system, to allow linkage to national databases.

HQIP are the data controller for NELA (hospitals in England and Wales) and a data sharing agreement is in place allowing sharing of pseudonymised NELA data for FLO-ELA participants as described in the participant consent materials. The FLO-ELA trial Sponsor is the data controller for the FLO-ELA data fields within the NELA database, for the data within the eCRF database used in Scotland and Northern Ireland, and for the data held within the randomisation system. FLO-ELA participant identifiable data held in the trial randomisation system will be linked to national databases to obtain outcome data including mortality and hospital (re)admission. Outcomes data will be merged with pseudonymised NELA/eCRF data to allow statistical and health economic analyses. Data sharing agreements will be established with NHS Digital and devolved nation equivalents.

7.7.1 Randomisation data

- NHS (England), CHI (Scotland) or H&S (Northern Ireland) number*
- Date of birth*
- Gender*

- Postcode*
- NELA ID (or assigned eCRF ID in Scotland and Northern Ireland)
- Checklist to ensure the patient meets the eligibility criteria
- Patient age
- ASA score
- Indication for planned surgery
- Centre ID (collected automatically during log-in to randomisation system)

*patient identifiers are collected to allow follow up of all randomised patients. See: section 7.10.

7.7.2 NELA dataset

The full list of data collected by NELA is in the NELA Participant Manual available at http://nela.org.uk/Audit-info-Documents#pt. The full data field list and subset of data fields that are shared with FLO-ELA (mirrored in the eCRF used in Scotland/Northern Ireland) are shown in Appendix 2.

Peri-operative data include: Date of birth, gender, date/time of hospital admission and other key aspects of pre-operative care, urgency and indication for surgery, patient risk scores and physiology markers, seniority of surgeon and anaesthetist during surgery, operative findings and procedure performed

Outcomes data include: Duration of stay in level 3 or level 2 bed, vital status at discharge.

7.7.3 Supplementary data fields for FLO-ELA

Intervention patients:

Intra-operative:

- Cardiac output monitoring (COM): time started / monitor type
- Number of cycles through protocol (number of COM-guided fluid boluses given)
- Total volume of crystalloid, colloid, blood and blood product administered
- Inotrope/vasopressor administered – type and mode of administration (bolus / infusion)

During the six hours after surgery:

- Cardiac output monitoring (COM): time started and stopped / monitor type
- Number of cycles through protocol (number of COM-guided fluid boluses given)
- Total volume of crystalloid, colloid, blood and blood product administered
- Inotrope/vasopressor administered – type and mode of administration (bolus / infusion)
- Duration of trial intervention (if <6 hours, reason for early termination: transfer to level 1 care area / other)

Control group patients:

Intra-operative:

- Total volume of crystalloid, colloid, blood and blood product administered
- Inotrope/vasopressor administered – type and mode of administration (bolus / infusion)
- Cardiac output monitoring used in a control patient? If yes: time started and indication (patient deterioration / other reason)

During the six hours after surgery:

- Total volume of crystalloid, colloid, blood and blood product administered
- Inotrope/vasopressor administered – type and mode of administration (bolus / infusion)
- Cardiac output monitoring used in a control patient? If yes: time started and indication (patient deterioration / other reason)
- Duration of trial intervention (if <6 hours, reason for early termination: transfer to level 1 care area / other)

7.7.4 Outcomes data from NHS Digital or devolved nation NHS datasets

- Mortality at 90 days and one year (via the ONS/NSSISD)
- Duration of the index hospital admission (HES/NSSISD/PEDW)
- Readmission to hospital as an inpatient (overnight stay) within 90 days and within one year of randomisation (HES/NSSISD/PEDW)

7.8 Predefined protocol deviations

- Failure to use cardiac output monitoring in an intervention group patient
- Failure to follow the haemodynamic algorithm (defined as at least one cycle of fluid bolus with measurement of stroke volume response) in an intervention group patient when a cardiac output monitor is being used.
- Use of cardiac output monitoring in a control group patient, including forms of monitoring based on stroke volume variation or pulse pressure variation only.

7.9 Follow-up procedures

Local investigators will review a participant's medical record (paper or electronic) in order to check and complete NELA/eCRF data entry. To collect data on the primary and secondary outcomes and enable the health economic analysis, we will request hospital episode statistics and mortality data from NHS Digital and equivalent devolved nation organisations. Prospective consent for data linkage will be sought before enrolment into the trial.

7.10 Withdrawal of participants

All study participants are free to withdraw from the study at any time. A participant withdrawal form will be completed for all participants withdrawn from the trial. However, ONS/HES (or equivalent) data will still be collected for these patients, and they will be included in the final analysis on an intention to treat basis, unless a participant specifically asks for their data not to be included (see section 8). In a small number of cases, after a patient has been randomised the patient may not ultimately undergo a surgical procedure in line with NELA inclusion criteria. This may be due to a change in clinical condition before the start of surgery such that no surgery is performed, due to a change in the surgery performed or due to unexpected findings during surgery. These patients are currently not included in the NELA dataset, and any data already entered to NELA is removed. Of these patients:

- Patients who do not ultimately undergo surgery will **not** be included in the analysis (see Section 5.9 on estimand framework). A trial withdrawal form should be completed for these patients.
- Patients who do undergo surgery **will** be included in the analysis, as long as they were *expected* to undergo an eligible NELA procedure at the time of randomisation. If it is thought to be clinically appropriate by the attending clinicians, trial treatment should continue in accordance with allocated group.

7.11 End of study definition

The end of the study is defined as the point when the last patient has completed one year follow-up. The Data Monitoring and Ethics Committee (DMEC) will monitor safety data throughout the trial. Based on these results, they could recommend termination of the trial on safety grounds. They will report any concerns to the Trial Steering Committee (TSC), who will inform the Sponsor and take appropriate action, which may include stopping the trial, to address concerns about participant safety. The Research Ethics Committee will be informed in writing if the trial is suspended or terminated early.

7.12 Schedule of assessment

Event/Visit	Screening	Pre-op	Intra-op	24 hrs post-op	Hospital discharge	Post-op day 90	Post-op 365 days
Inclusion/exclusion criteria	x						
Informed consent	x						
NELA/eCRF pre-operative data*		x					
Randomisation		x					
NELA/eCRF intraoperative data*			x	x			
Fluid and inotropic therapy			x	x			
NELA/eCRF postoperative data by medical notes review*				x	x		
NELA/eCRF duration of hospital stay and days in critical care*					x		

SAE				x	x	x	x
Hospital readmissions (HES)						x	X
Vital status (ONS)						x	x
End of trial form							x

****these data are already collected as routine care by medical teams for NELA in England and Wales***

8 STATISTICAL CONSIDERATIONS

8.1 Sample size calculation

The original sample size calculation was based on the original primary outcome of mortality within 90 days of randomisation. Based on a 5% alpha level, 90% power, and assuming a 2% dropout rate and a 19% 90-day mortality rate in the usual care arm, 3823 patients were required in each arm (7646 total) to detect a risk ratio of 0.85 (equivalent to an absolute decrease from 19% to 16.15%) for the primary outcome.

For the new primary outcome of DAOH-90, we calculated that 3138 participants (1569 per group) would be required to detect a 3.2-day increase in DAOH-90 (from mean 64.5 (SD 28.0) days in the control group to 67.7 (SD 27.1) days in the intervention group), with 90% power, a 5% alpha (Type I error) level, and a 2% dropout rate.

The revised sample size calculation was made without access to or knowledge of unblinded data. The parameter choices for this sample size calculation were derived from simulation as detailed in the trial Statistical Analysis Plan V4.0. In summary:

Mortality

Patients who died within 90 days are assigned a DAOH-90 value of 0 (18). Prior to the revised sample size calculation based on the DAOH-90 primary outcome, pooled (control and intervention group combined) 90-day mortality was reviewed on the advice of the FLO-ELA Data Monitoring Committee in September 2019 and found to be ~12%. Therefore, the assumed control-arm event was set at ~13%, and as per the original sample size calculation, we assumed the intervention decreased mortality by 15% (relative risk 0.85) to ~11%.

Time spent in hospital

Previous studies of this intervention in higher risk (mortality >10% at longest follow-up) patient populations have found reductions in postoperative length of stay (LoS) of 1.3 days (95% CI 0.1-2.5) (6,12–15,26–28). When also considering morbidity-related hospital readmissions, a mean 2-day difference in time spent in hospital for those surviving to 90-days is realistic.

Overall effect on DAOH-90

Using statistical simulation based on the above parameters and summary hospital length of stay data from NELA (November 2020), we estimated that a 2-day difference in time in hospital for survivors and a relative risk reduction for 90-day mortality in patients in the intervention arm of 0.85 would give expected mean [SD] DAOH-90 in the Control arm of ~ 64.5 days [28.0] and in the intervention arm ~ 67.7 days [27.1], i.e. an overall 3.2-day increase in DAOH-90 in the intervention group.

This proposed effect size is realistic and of clear impact to patients and healthcare systems, representing several hundred lives saved each year, and a mean of two days less time spent in hospital for survivors if the intervention is effective and fully implemented.

8.2 Statistical analysis

The number of patients recruited and followed up will be recorded in a CONSORT flow chart. Baseline characteristics will be summarised by treatment group.

Patients will be analysed according to the treatment group to which they were randomised and all eligible patients for whom an outcome is available will be included in the analysis (29) except (i) those randomised in error (i.e. were ineligible at the time of randomisation) and (ii) those who were eligible and randomised but did not ultimately undergo surgery as per the principal stratum strategy outlined in Table 1. The former exclusion (participants randomised in error) is based on pre-randomisation information (i.e. failure to meet the eligibility criteria) and as such will be unbiased. The latter exclusion (participants who did not undergo surgery) will be unbiased for the principal stratum effect under the assumption that treatment group allocation does not affect whether participants undergo surgery or not (i.e. a participant in the intervention group who does not undergo surgery would also not receive surgery had they been allocated to the control, and *vice versa*). This assumption is justified on the basis that, in most cases, the relevant decision makers will be unaware of trial group allocation until surgery starts (i.e. at the point the decision is made). Further, the decision not to proceed with surgery has large health implications for the patient, and is only undertaken in response to a major change in the patient's clinical condition since surgery was initially planned, and it is implausible that such a fundamental change in patient care would be undertaken on the basis of the planned method of fluid delivery.

For each analysis we will present the number of patients included in the analysis, a summary measure of the outcome in each treatment group, treatment effect, 95% confidence interval and a two-sided p-value. P-values <0.05 will be considered statistically significant.

The estimand for the primary outcome (days alive and out of hospital within 90 days of randomisation) will be estimated using a mixed-effects linear regression model, with a random-intercept for centre (30). The model will be adjusted for the minimisation factors of patient age and ASA class (I, II, III, IV, and V) (31), as well as the following prognostic baseline covariates: urgency of surgery (Immediate, Urgent, and Expedited), Glasgow Coma Score (GCS), systolic blood pressure and pulse rate (32). Urgency of surgery and ASA class will be included as categorical variables, while patient age, GCS, systolic blood pressure, and pulse rate will be included as continuous variables. Patient age and GCS will be including assuming

a linear association with the outcome, and systolic blood pressure and pulse rate will be included using restricted cubic splines with 3 knots (knots will be placed based on Harrell's recommended percentiles) (33,34). Missing baseline data will be handled using mean imputation for continuous variables, and a missing indicator variable for categorical variables (35). The estimands for the secondary outcomes will be estimated using an analogous mixed effects logistic regression model, with covariates and random effect specification as above and the same principal stratum strategy adopted.

We will also conduct subgroup analysis of the primary outcome by urgency of surgery (Immediate vs. Urgent vs. Expedited), age (<70 vs. 70+), indication for surgery (bowel perforation vs. bowel obstruction without perforation vs. other indications), gender (male vs. female) and a pre-operative predicted risk score, which will be determined, with a binary cut-off established for the subgroup analysis, before any of the data is viewed. In addition, we will carry out two analyses to assess the impact of the Covid-19 pandemic on treatment effect; the first will be a subgroup analysis based on whether patient was randomised pre or post Covid-19 pandemic (pre = prior to cut-off date 30 January 2020, post = 30 January 20 or after) and in the second analysis, subgroups will be Covid-19 status of patient (negative [0] or positive [1]), subject to test availability - this information has only been recorded systematically since March 2020. Subgroup analysis will be undertaken by statistically testing the interaction between treatment group and subgroup variable. In the case of subgroup variables age and preoperative risk score, this will involve in addition the fitting of cubic splines as described in SAP V4.0. Subgroup specific estimates and 95% confidence intervals will also be reported. Any subgroup findings will be treated with caution and will be given less weight than the primary analysis.

Days at home analysis: The revised primary outcome, DAOH-90, may be considered a proxy for days *at home* within 90 days (DAH-90). However, we will not have sufficiently detailed data to track each individual pathway in terms of residence outside of hospital for everyone in the database.

In order to assess if inference on DAOH-90 may be extended to DAH-90, we will analyse data for a subset of FLO-ELA participants for whom post-discharge destination ("home" or "residence other than own home") is recorded. This was recorded as part of NELA audit up to December 2019, but not thereafter. DAH-90 will be calculated in the same way as DAOH-90, except that in instances where a patient is discharged to residence other than own home, DAH-90 will be set to zero. The primary analysis on DAOH-90 will be repeated with DAH-90 for patients with available data. This will be compared against results of the primary analysis on DAOH-90 for (i) all patients (ii) subset of patients on which DAH-90 analysis carried out.

The same overarching analysis strategy will be applied with respect to all sub-group analyses and for the days-at-home analysis as that described for the primary estimand.

We will in addition present summary statistics on NELA-defined treatment standards and the pre-surgical risk profile of FLO-ELA participants according to whether the patient was randomised in the pre or post Covid pandemic era, using the same cut-off date as for the treatment effect analysis, i.e. prior to (pre) or on or after (post) 30 January 2020. The cut-off date of 30th January 2020 was chosen as this was when Covid-19 was first confirmed to be present in the UK.

8.3 Health economic analysis

The economic evaluation would adhere, as far as possible, to the most up to date NICE Guide to the Methods of Technology Appraisal (36) to ensure that trial findings are informative for national-level policy considerations. The perspective will be limited to NHS secondary care, which will likely cover the main drivers of total care costs, including the initial hospital admission (including the treatment of complications) and subsequent hospital (re)admissions during the 90-days and, separately, during one year from randomisation. NELA/eCRF will provide all relevant individual-level resource use information related to the initial hospital admission, including that related to implementation of the intervention. Electronic hospital resource use data (including inpatient, outpatient and critical care episodes) will be obtained from NHS Digital (or devolved nation equivalents) for the one-year period post-randomisation to estimate total secondary care cost over the 90-day and one year follow-up period. In addition, hospital data covering a period of 90 days pre-randomisation will be retrospectively obtained from NHS Digital (or equivalent) in order to carry out adjustments for baseline differences in mean cost between comparison groups.

Unit costs for each assessed resource use item will be collated from national sources (such as the NHS Reference Costs) where possible; adjustments and additional estimates will be obtained from published studies and expert opinion where needed. These will be applied to individual-level resource use to estimate individual-level costs.

Due to the lack of direct patient assessments in this trial, quality-adjusted life years (QALYs) will be estimated from EQ5D-3L assessments in the EPOCH trial (37) using a mapping approach relevant to the available data (to be determined and established prior to the health economics analysis plan being signed off). Broadly, this will involve estimating an EQ-5D-3L or tariff prediction model in the EPOCH data using relevant patient characteristics common to both studies, and applying that model to our study data to predict EQ-5D-3L or tariff values.

The comparison of resulting QALYs and costs will broadly follow the outcomes analyses (e.g. same comparison groups, intention-to-treat basis, adjustment for minimisation factors and other pre-specified

covariates) and bootstrapped regression approaches (5000 replications) will be used to evaluate uncertainty in the results.

The economic analyses will include cost-effectiveness analyses, measured in terms of incremental cost per DAOH at 90 days (DAOH-90) and incremental cost per QALY gained at two time periods of follow-up: at 90 days and at one year post randomisation. Incremental cost-effectiveness ratios will be reported.

Uncertainty around the cost-effectiveness ratios will be analysed using a bootstrap approach (5000 replications) and summarised using cost-effectiveness planes and cost-effectiveness acceptability curves (CEACs) over a range of relevant values that decision-makers may place upon outcome improvements (λ). (For QALYs the range is likely to be £0 to £50,000 to include the current value placed on QALY gains in decision-making by NICE). For each value of λ, the proportion of iterations indicating a higher net benefit for the intervention arm will be calculated and plotted as a CEAC.

Sensitivity analyses will be used to examine the effect of analytic assumptions on the results of the cost-effectiveness and cost-utility analyses. This will include the unit costs used to value intervention cost. All cost, outcome and unit cost data used for the economic evaluation will also be presented in a disaggregated format to facilitate interpretations from alternative perspectives.

A health economics analysis plan, specifying in detail the health economics analyses, will be finalised and signed off prior to unblinding the team analysing the study. Longer-term extrapolation will be considered in case of remaining policy uncertainty (e.g. evidence for survival benefit but unclear cost-effectiveness within one year post randomisation), to project survival of participants and evaluate the cost-effectiveness of cardiac output-guided fluid therapy over a longer time period.

8.4 Secondary studies

The use of FLO-ELA trial data for further secondary studies is encouraged. A prospective statistical analysis plan will be prepared for each secondary study before data analysis commences.

9 RESEARCH ETHICS

The PI will ensure that this trial is conducted in accordance with the Principles of the Declaration of Helsinki as amended in Tokyo (1975), Venice (1983), Hong Kong (1989), South Africa (1996), Edinburgh (2000), Washington DC (2002), Tokyo (2004), Seoul (2008) and Fortaleza (2013) as described at the following internet site: http://www.wma.net/en/30publications/10policies/b3/index.html. The trial will fully adhere to the principles outlined in the Guidelines for Good Clinical Practice ICH Tripartite Guideline (January 1997) and to the Mental Capacity Act 2005. The study will be carried out in accordance with the ethical principles in the Research Governance Framework for Health and Social Care, Second Edition, 2005 and its

subsequent amendments as applicable and applicable legal and regulatory requirements. At sites, all accompanying material given to a potential participant will have undergone an independent Research Ethics Committee review. Full approval by the Research Ethics Committee will be obtained prior to starting the trial and fully documented by letter to the Chief Investigator naming the trial site, local PI (who may also be the Chief Investigator) and the date on which the ethics committee deemed the trial as permissible at that site. All members of the trial steering committee will declare conflicts of interest before joining the study group. These will be listed on any publications arising from the trial.

10 DATA HANDLING AND RECORD KEEPING

10.1 Confidentiality

Information related to participants will be kept confidential and managed in accordance with the Data Protection Act (UK), NHS Caldicott Principles (UK), The Research Governance Framework for Health and Social Care (UK), and the conditions of Research Ethics Committee Approval, or corresponding legislation or approvals for a particular participating site. The patient's NHS Number, gender, date of birth and postcode will be collected at randomisation to allow tracing through national records. The personal data recorded on all documents will be regarded as confidential. The PI must maintain in strict confidence trial documents, which are to be held in the local hospital (e.g. patients' written consent forms). The PI must ensure the patient's confidentiality is maintained at all times. The Sponsor will ensure that all participating partner organisations will maintain the confidentiality of all subject data and will not reproduce or disclose any information by which subjects could be identified, other than reporting of serious adverse events. Representatives of the trial management team will require access to patient notes for quality assurance purposes and source data verification, but patients should be reassured that their confidentiality will be respected at all times. In the case of special problems and/or competent authority queries, it is also necessary to have access to the complete trial records, provided that patient confidentiality is protected.

10.2 Data storage

Data will be entered directly on to the secure NELA data entry web portal (in England and Wales) or equivalent mirror eCRF database for Scotland and Northern Ireland, and on to the secure trial randomisation system. Submitted data will be reviewed for completeness and consistency by authorised users within the study group. Submitted data will be stored securely against unauthorised manipulation and accidental loss since only authorised users at site, the Sponsor organisation, Queen Mary University of London or NELA (host of the data entry portal) will have access. Desktop security is maintained through user names and passwords. Data back-up procedures are in place. Storage and handling of confidential trial data and documents will be in accordance with the Data Protection Act 2018 (UK) and General Data Protection Regulation.

10.3 Archiving

Each site will maintain and securely store an investigator site file. NELA and the PCTU (QMUL) will be responsible for archiving identifiable data. Data will be archived in accordance with local standards and procedures for quality and assurance. All other trial documentation and data will be archived by the Sponsor and PCTU in a purpose designed archive facility for twenty years in accordance with regulatory requirements. Access to these archives will be restricted to authorised personnel. Electronic data sets will be stored indefinitely.

10.4 Patient identifiable data

To facilitate linkage to national databases for the collection of follow-up data, patient identifiable data will be collected and entered on to the secure NELA data entry web portal and the randomisation system. Data will be stored and handled in accordance with the Data Protection Act 2018 (UK) and General Data Protection Regulation or equivalent legislation for a particular country or site. In the event that patient identifiable data needs to be transferred between authorised users, this will occur by email from @nhs.net to @nhs.net.

11 PRODUCTS, DEVICES AND TECHNIQUES

11.1 Cardiac output-guided haemodynamic therapy

Cardiac output monitors are routinely used in secondary care. Investigators may only use commercially available cardiac output monitoring equipment shown to accurately track changes in cardiac stroke volume, from a list of devices in the intervention SOP. Please see the study intervention SOP for specific details of the intervention.

12 SAFETY REPORTING

12.1 Adverse Events (AE)

An AE is any untoward medical occurrence in a subject to whom an intervention has been administered, including occurrences which are not necessarily caused by or related to that intervention. An AE can therefore be any unfavourable and unintended sign (including an abnormal laboratory finding), symptom or disease temporarily associated with study activities. However, FLO-ELA is a non-CTIMP trial, and all trial interventions are already in routine clinical use for patients undergoing emergency laparotomy surgery. Furthermore, adverse events are very common following emergency laparotomy. AEs will therefore not be collected for the FLO-ELA trial. The DMEC will monitor the safety of the intervention by reviewing in-hospital mortality rates and reported SAEs at intervals in both trial groups.

12.2 Serious Adverse Event (SAE)

A serious adverse event (SAE) is defined as an untoward occurrence that:

(a) results in death;
(b) is life-threatening;
(c) requires hospitalisation or prolongation of existing hospitalisation;
(d) results in persistent or significant disability or incapacity;

An SAE occurring to a research participant should be reported to the sponsor where in the opinion of the Principal Investigator the event was:

- Related – that is, it resulted from administration of any of the research procedures, and
- Unexpected – that is, the type of event is not listed in the protocol as an expected occurrence.

The FLO-ELA trial is an investigation of a perioperative intervention. It is expected that patients undergoing emergency laparotomy surgery will suffer medical complications, with consequences up to and including death. Only complications considered by the local PI or delegated authority (blinded to study group allocation) to be *related to the use of study procedures* and not a typical complication of emergency bowel surgery should be reported as SAEs. Typical complications of emergency bowel surgery, which should not be reported as SAEs, are included in Appendix 4.

12.3 Notification and reporting of Serious Adverse Events

Serious Adverse Event (SAEs) that are considered to be 'related' and 'unexpected' are to be reported to the sponsor and the sponsor's representative within 24 hours of learning of the event. The event will be reviewed by the CI and if it meets the criteria for an SAE, will then be reported to the REC within 15 days of receipt of the SAE report.

12.4 Reporting a Serious Adverse Event

The local PI or delegated authority will be blinded to study group allocation and will review any reported events to ensure that they meet the criteria for an SAE. Individual sites will notify the co-ordinating centre in that country of an SAE by emailing a scanned copy of the supplementary SAE report form to the national co-ordinator. An SAE log should be maintained at site to record the details of the SAE and its review until resolution. SAEs will be reported within 24 hours and will be forwarded to the sponsor via the co-ordinating centre.

12.5 Urgent safety measures

The CI may take urgent safety measures to ensure the safety and protection of trial participants from any immediate hazard to their health and safety. The measures should be taken immediately. In this instance, the approval of the REC prior to implementing these safety measures is not required. However, it is the

responsibility of the CI to inform the sponsor and Research Ethics Committee of this event in writing, setting out the reasons for the urgent safety measures and the plan for further action, within three days. The sponsor must be sent a copy of the correspondence with regards to this matter.

12.6 Annual safety reporting

The CI will send the annual progress report to the REC and to the sponsor starting 12 months after the date of the favourable opinion.

12.7 Overview of the safety reporting responsibilities

The CI/PI has the overall oversight responsibility. The CI/PI will ensure that safety monitoring and reporting is conducted in accordance with the sponsor's requirements.

13 MONITORING & AUDITING

The Sponsor and PCTU will have oversight of the trial conduct at each site. The trial team will take day-to-day responsibility for ensuring compliance with the requirements of GCP in terms of quality control and quality assurance of the data collected as well as safety reporting. The FLO-ELA Trial Management Group will communicate closely with individual sites and the Sponsor's representatives to ensure these processes are effective. A Data Monitoring and Ethics Committee (DMEC) has been appointed (see section 14.3).
The PCTU quality assurance manager has conducted a study risk assessment in collaboration with the CI. Based on the risk assessment, an appropriate study monitoring and auditing plan has been produced according to PCTU SOPs. Any changes to the monitoring plan must be agreed by the PCTU QA manager and C.I.

13.1 Monitoring the safety and wellbeing of trial participants

The Research and Development departments at each trial site should perform regular audits of research practice. Systems are in place to ensure that all PIs and designees are able to demonstrate that they are qualified by education, training or experience to fulfill their roles and that procedures are in place that assures the quality of every aspect of the trial. The intervention will last less than 12 hours in most cases, therefore it is extremely unlikely that new safety information will arise during the intervention period. Nonetheless should this situation arise, participants will be informed and asked if they wish to discontinue the intervention. If the subjects wish to continue in the trial they will be formally asked to sign a revised approved patient information sheet and consent form. Early termination of trial in response to safety issues will be addressed via the DMEC. Day to day management and monitoring of individual sites will be undertaken via the Trial Management Group composed of the Chief Investigator and supporting staff. They will meet on a regular basis to discuss trial issues.

13.2 *Monitoring the safety of investigators*

Each site has health and safety policies for employees. All personnel should ensure that they adhere to health and safety regulations relating to their area of work. The PI will ensure that all personnel have been trained appropriately to undertake their specific tasks. The trial team will complete GCP and consent training prior to start up.

14 TRIAL MANAGEMENT & COMMITTEES

14.1 *Trial management group*

Day-to-day trial management will be co-ordinated by a trial management group consisting of the Chief Investigator, their support staff and members of the PCTU.

14.2 *Trial steering committee*

The TSC has been formed in accordance with NIHR guidance. It will oversee the trial and consists of:

- several independent clinicians, statistician and trialists
- lay representation
- co-investigators
- an independent Chair

Meetings will be held at regular intervals determined by need but not less than once a year. The TSC will take responsibility for:

- approving the final trial protocol;
- major decisions such as a need to change the protocol for any reason;
- monitoring and supervising the progress of the trial;
- reviewing relevant information from other sources;
- considering recommendations from the DMEC and
- informing and advising on all aspects of the trial

14.3 *Data monitoring and ethics committee*

The Data Monitoring and Ethics Committee (DMEC) has been formed in accordance with NIHR guidance. It is independent of the trial team and comprises of two clinicians with experience in undertaking clinical trials and a statistician. The committee will agree conduct and remit, which will include the early termination process. During the period of recruitment into the trial the DMEC will monitor safety data and routinely meet to assess safety analyses. The trial will be terminated early if there is evidence of harm in the intervention group or if recruitment is futile. The DMEC functions primarily as a check for safety by reviewing SAEs and in-hospital mortality.

15 FINANCE AND FUNDING

The FLO-ELA trial is funded by the NIHR Health Technology Assessment Programme (15/80/54). The views expressed are those of the author(s) and not necessarily those of the NIHR or the Department of Health and Social Care.

16 SPONSORSHIP & INDEMNITY

University Hospital Southampton NHS Foundation Trust will act as Sponsor and provide no fault insurance.

17 PUBLICATION

Data arising from this research will be made available to the scientific community in a timely and responsible manner. A detailed scientific report will be submitted to a widely accessible scientific journal on behalf of the FLO-ELA Trial Steering Committee. The TSC will agree the membership of a writing committee, which will take primary responsibility for final data analysis and writing of the scientific report. All members of the writing committee will comply with internationally agreed requirements for authorship and will approve the final manuscript prior to submission. Please see FLO-ELA trial publication charter for further details.

18 REFERENCES

1. NELA project team. First Patient Report of the National Emergency Laparotomy Audit. Royal College of Anaesthetists; 2015.

2. NELA project team. The Second Patient Report of the National Emergency Laparotomy Audit [Internet]. 2016. Available from: http://nela.org.uk/reports

3. Cannesson M, Pestel G, Ricks C, Hoeft A, Perel A. Hemodynamic monitoring and management in patients undergoing high risk surgery: a survey among North American and European anesthesiologists. Crit Care. 2011;15(4):R197.

4. Jhanji S, Vivian-Smith A, Lucena-Amaro S, Watson D, Hinds CJ, Pearse RM. Haemodynamic optimisation improves tissue microvascular flow and oxygenation after major surgery: a randomised controlled trial. Crit Care. 2010;14(4):R151.

5. Bangash MN, Patel NS, Benetti E, Collino M, Hinds CJ, Thiemermann C, et al. Dopexamine can attenuate the inflammatory response and protect against organ injury in the absence of significant effects on hemodynamics or regional microvascular flow. Crit Care. 2013 Mar 28;17(2):R57.

6. Pearse RM, Harrison DA, MacDonald N, Gillies MA, Blunt M, Ackland G, et al. Effect of a perioperative, cardiac output-guided hemodynamic therapy algorithm on outcomes following major gastrointestinal surgery: a randomized clinical trial and systematic review. JAMA. 2014 May 19;311(21):2181–90.

7. Shapter SL, Paul MJ, White SM. Incidence and estimated annual cost of emergency laparotomy in England: is there a major funding shortfall? Anaesthesia. 2012 May;67(5):474–8.

8. The ProCESS Investigators. A randomized trial of protocol-based care for early septic shock. N Engl J Med. 2014 Mar 18;370:1683–93.

9. ARISE Investigators, ANZICS Clinical Trials Group, Peake SL, Delaney A, Bailey M, Bellomo R, et al. Goal-directed resuscitation for patients with early septic shock. N Engl J Med. 2014 Oct 16;371(16):1496–506.

10. Power GS, Harrison DA, Mouncey PR, Osborn TM, Harvey SE, Rowan KM. The Protocolised Management in Sepsis (ProMISe) trial statistical analysis plan. Crit Care Resusc. 2013 Dec;15(4):311–7.

11. Harten J, Crozier JEM, McCreath B, Hay A, McMillan DC, McArdle CS, et al. Effect of intraoperative fluid optimisation on renal function in patients undergoing emergency abdominal surgery: a randomised controlled pilot study (ISRCTN 11799696). Int J Surg. 2008 Jun;6(3):197–204.

12. Boyd O, Grounds RM, Bennett ED. A randomized clinical trial of the effect of deliberate perioperative increase of oxygen delivery on mortality in high-risk surgical patients. JAMA. 1993 Dec 8;270(22):2699–707.

13. Pearse R, Dawson D, Fawcett J, Rhodes A, Grounds RM, Bennett ED. Early goal-directed therapy after major surgery reduces complications and duration of hospital stay. A randomised, controlled trial [ISRCTN38797445]. Critical Care. 2005;9(6):R687–93.

14. Sandham JD, Hull RD, Brant RF, Knox L, Pineo GF, Doig CJ, et al. A randomized, controlled trial of the use of pulmonary-artery catheters in high-risk surgical patients. N Engl J Med. 2003 Jan 2;348(1):5–14.

15. Shoemaker WC, Appel PL, Kram HB, Waxman K, Lee TS. Prospective trial of supranormal values of survivors as therapeutic goals in high-risk surgical patients. Chest. 1988 Dec;94(6):1176–86.

16. Buggy DJ, Freeman J, Johnson MZ, Leslie K, Riedel B, Sessler DI, et al. Systematic review and consensus definitions for standardised endpoints in perioperative medicine: postoperative cancer outcomes. British Journal of Anaesthesia. 2018 Jul 1;121(1):38–44.

17. Moonesinghe SR, Jackson AIR, Boney O, Stevenson N, Chan MTV, Cook TM, et al. Systematic review and consensus definitions for the Standardised Endpoints in Perioperative Medicine initiative: patient-centred outcomes. British Journal of Anaesthesia. 2019 Nov 1;123(5):664–70.

18. Jerath A, Austin PC, Wijeysundera DN. Days Alive and Out of HospitalValidation of a Patient-centered Outcome for Perioperative Medicine. Anesthesiology. 2019 Jul 1;131(1):84–93.

19. Pinkney TD, Calvert M, Bartlett DC, Gheorghe A, Redman V, Dowswell G, et al. Impact of wound edge protection devices on surgical site infection after laparotomy: multicentre randomised controlled trial (ROSSINI Trial). BMJ. 2013;347:1–13.

20. Kay R, Siriwardena A. The process of informed consent for urgent abdominal surgery. J Med Ethics. 2001 Jun;27(3):157–61.

21. Deans KJ, Minneci PC, Danner RL, Eichacker PQ, Natanson C. Practice Misalignments in Randomized Controlled Trials: Identification, Impact and Potential Solutions. Anesth Analg. 2010 Aug;111(2):444–50.

22. NICE. Intravenous fluid therapy in adults in hospital (Clinical Guidance 174) [Internet]. 2014 [cited 2014 Apr 16]. Available from: http://www.nice.org.uk/

23. Gray LD, Morris C. The principles and conduct of anaesthesia for emergency surgery. Anaesthesia. 2013 Jan;68 Suppl 1:14–29.

24. Stoneham M, Murray D, Foss N. Emergency surgery: the big three – abdominal aortic aneurysm, laparotomy and hip fracture. Anaesthesia. 2014 Jan 1;69:70–80.

25. Kahan BC, Rehal S, Cro S. Risk of selection bias in randomised trials. Trials. 2015;16:405.

26. Calvo-Vecino JM, Ripollés-Melchor J, Mythen MG, Casans-Francés R, Balik A, Artacho JP, et al. Effect of goal-directed haemodynamic therapy on postoperative complications in low–moderate risk surgical patients: a multicentre randomised controlled trial (FEDORA trial). British Journal of Anaesthesia [Internet]. 2018 Feb [cited 2018 Mar 11]; Available from: http://linkinghub.elsevier.com/retrieve/pii/S0007091217542075

27. Lobo SM, Salgado PF, Castillo VG, Borim AA, Polachini CA, Palchetti JC, et al. Effects of maximizing oxygen delivery on morbidity and mortality in high-risk surgical patients. Crit Care Med. 2000 Oct;28(10):3396–404.

28. Wilson J, Woods I, Fawcett J, Whall R, Dibb W, Morris C, et al. Reducing the risk of major elective surgery: randomised controlled trial of preoperative optimisation of oxygen delivery. BMJ. 1999 Apr 24;318(7191):1099–103.

29. White IR, Horton NJ, Carpenter J, statistics reader in medical and social, Pocock SJ. Strategy for intention to treat analysis in randomised trials with missing outcome data. BMJ. 2011;342:d40.

30. Kahan BC. Accounting for centre-effects in multicentre trials with a binary outcome - when, why, and how? BMC Med Res Methodol. 2014;14:20.

31. Kahan BC, Morris TP. Improper analysis of trials randomised using stratified blocks or minimisation. Stat Med. 2012 Feb 20;31(4):328–40.

32. Kahan BC, Jairath V, Doré CJ, Morris TP. The risks and rewards of covariate adjustment in randomized trials: an assessment of 12 outcomes from 8 studies. Trials. 2014;15:139.

33. Kahan BC, Rushton H, Morris TP, Daniel RM. A comparison of methods to adjust for continuous covariates in the analysis of randomised trials. BMC Med Res Methodol. 2016;16:42.

34. Harrell FE. Regression Modeling Strategies [Internet]. New York, NY: Springer New York; 2001 [cited 2016 Jul 21]. (Springer Series in Statistics). Available from: http://link.springer.com/10.1007/978-1-4757-3462-1

35. White IR, Thompson SG. Adjusting for partially missing baseline measurements in randomized trials. Stat Med. 2005 Apr 15;24(7):993–1007.

36. National Institute for Health and Care Excellence. Guide to the methods of technology appraisal 2013 (PMG9) [Internet]. 2013. 93 p. Available from: nice.org.uk/process/pmg9

37. Peden CJ, Stephens T, Martin G, Kahan BC, Thomson A, Rivett K, et al. Effectiveness of a national quality improvement programme to improve survival after emergency abdominal surgery (EPOCH): a stepped-wedge cluster-randomised trial. Lancet. 2019 Apr 25;

Appendix 1: NELA Inclusion & Exclusion Criteria (version 1.7, dated 24/02/2017)

NELA Inclusion Criteria

NELA will enrol the patients treated in England or Wales who meet the following criteria:

- aged 18 years and over
- who undergo an expedited, urgent or emergency (NCEPOD definitions) abdominal procedure on the gastrointestinal tract

This will include:

- Open, laparoscopic, or laparoscopically-assisted procedures
- Procedures involving the stomach, small or large bowel, or rectum for conditions such as perforation, ischaemia, abdominal abscess, bleeding or obstruction
- Washout/evacuation of intra-peritoneal abscess (unless due to appendicitis or cholecystitis - excluded, see below)
- Washout/evacuation of intra-peritoneal haematoma
- Bowel resection/repair due to incarcerated incisional, umbilical, inguinal and femoral hernias (but not hernia repair without bowel resection/repair) E.g. large incisional hernia repair with bowel resection.
- Bowel resection/repair due to obstructing/incarcerated incisional hernias provided the presentation and findings were acute. This will include large incisional hernia repair with division of adhesions.
- Laparotomy/laparoscopy with inoperable pathology (e.g. peritoneal/hepatic metastases) where the intention was to perform a definitive procedure. This does not include purely diagnostic procedures.
- Laparoscopic/open adhesiolysis
- Return to theatre for repair of substantial dehiscence of major abdominal wound (i.e. "burst abdomen")
- Any reoperation/return to theatre for complications of elective general/upper gastrointestinal surgery meeting the criteria above is included. Returns to theatre for complications following non-GI surgery are now excluded (see exclusion criteria below).

If multiple procedures are performed on different anatomical sites within the abdominal/pelvic cavity, the patient would be included if the major procedure is general surgical. E.g.

- Non-elective colonic resection with hysterectomy for a fistulating colonic cancer would be included as the bowel resection is the major procedure
- However bowel resection at the same time as emergency abdominal aortic aneurysm repair would not be included as the aneurysm repair is the major procedure

The above criteria are not exhaustive. The NELA team should be contacted if any clarification is required.

NELA Exclusion Criteria

Patients with the following characteristics will be excluded from NELA:

1. Patients under 18
2. Elective laparotomy / laparoscopy
3. Diagnostic laparotomy/laparoscopy where no subsequent procedure is performed (NB, if no procedure is performed because of inoperable pathology, then include)
4. Appendicectomy +/- drainage of localised collection unless the procedure is incidental to a non-elective procedure on the GI tract
5. Cholecystectomy +/- drainage of localised collection unless the procedure is incidental to a non-elective procedure on the GI tract (All surgery involving the appendix or gallbladder, including any surgery relating to complications such as abscess or bile leak is excluded. The only exception to this is if carried out as an incidental procedure to a more major procedure. We acknowledge that there might be extreme cases of peritoneal contamination, but total exclusion avoids subjective judgement calls about severity of contamination.)
6. Non-elective hernia repair without bowel resection or division of adhesions
7. Minor abdominal wound dehiscence unless this causes bowel complications requiring resection
8. Non-elective formation of a colostomy or ileostomy as either a trephine or a laparoscopic procedure (NB: if a midline laparotomy is performed, with the primary procedure being formation of a stoma then this should be included)
9. Vascular surgery, including abdominal aortic aneurysm repair
10. Caesarean section or obstetric laparotomies
11. Gynaecological laparotomy
12. Ruptured ectopic pregnancy, or pelvic abscesses due to pelvic inflammatory disease
13. Laparotomy/laparoscopy for pathology caused by blunt or penetrating trauma
14. All surgery relating to organ transplantation (including returns to theatre for any reason following transplant surgery)
15. Surgery relating to sclerosing peritonitis
16. Surgery for removal of dialysis catheters
17. Laparotomy/laparoscopy for oesophageal pathology
18. Laparotomy/laparoscopy for pathology of the spleen, renal tract, kidneys, liver, gall bladder and biliary tree, pancreas or urinary tract
19. Returns to theatre for complications (e.g. bowel injury, haematoma, collection) following non-GI surgery are now excluded i.e. returns to theatre following renal, urological, gynaecological, vascular, hepatic, pancreatic, splenic surgery are excluded.

Appendix 2: NELA data entry

Data fields relating exclusively to FLO-ELA participants are only visible for NELA centres participating in the FLO-ELA trial and are marked in blue in the table below. These data fields are only activated depending on the responses to fields 4.4 and 4.F.2

1.	Demographics and Admission	Format	Notes
19 1.1	20 NHS Number		**Not shared with FLO-ELA**
**1.2**	Pseudo-anonymisation		Computer generated “NELA ID”
1.3	Local patient id/hospital number		**Not shared with FLO-ELA**
1.4	Date of birth		**Not shared with FLO-ELA**
	Age on arrival		**Not shared with FLO-ELA**
1.5	Sex	▪ Male / ▪ Female	**Not shared with FLO-ELA**
1.6	Forename		**Not shared with FLO-ELA**
1.7	Surname		**Not shared with FLO-ELA**
1.8	Postcode		**Not shared with FLO-ELA**
**1.9**	Date and time the patient first arrived at the hospital/Emergency department		Admission time is 1st presentation to hospital/A&E. If the GP out of hours centre is based at the hospital A&E, then use time care was transferred from GP to the hospital. I.e. Admission time is intended to reflect the time at which the patient's care became the responsibility of the hospital.
1.10	What was the nature of this admission?	▪ Elective / ▪ Non-elective	No longer collected in NELA from 1/12/19
1.10b	If non-elective, what was the initial route of admission/assessment?	○ Assessed initially in Emergency Department<br>○ Assessed initially in “front of house” acute surgical assessment unit<br>○ Direct referral to ward by GP	No longer collected in NELA from 1/12/19
1.11	Which specialty was this patient first admitted under?	○ General surgery<br>○ General medicine<br>○ Gastroenterology<br>○ Elderly Care<br>○ Other	No longer collected in NELA from 1/12/19
1.12	Residence before this hospital admission	○ Own home/sheltered housing<br>○ Residential care<br>○ Nursing care<br>○ Unknown	No longer collected in NELA from 1/12/19

**1.13a**	Is this patient known to have a Learning Disability?	○	**Not shared with FLO-ELA** (introduced since start of trial recruitment) No longer collected in NELA from 1/12/2020
**1.13b**	Is this patient known to have an Autistic Spectrum Disorder?	○	**Not shared with FLO-ELA** (introduced since start of trial recruitment)<br>No longer collected in NELA from 1/12/2020

**2**	**Pre-op**	**Format**	**Notes**
**If the patient is returning to theatre as an emergency following previous elective surgery, all answers should relate to the emergency laparotomy, not the previous elective surgery.**			
**2.1**	Date and time first seen by consultant surgeon following admission with acute abdomen. If under care of a non-surgical specialty, this should be the time 1st seen after referral to general surgeons..	Date ____________(DD/MM/YYYY)<br>○ Date not known<br>Time_____________ (HH:MM)<br>○ Time not known<br>○ Not Seen	No longer collected in NELA from 1/12/19
**2.2**	Date and time that the decision was made to operate<br>*If this is unavailable please enter date and time that this patient was first booked for theatre for emergency laparotomy*	Date ____________(DD/MM/YYYY)<br>○ Date not known<br>Time_____________ (HH:MM)<br>○ Time not known	If the time is unknown for "decision made", but date and time known for "booking", please provide full details of the latter. If only date is known for both fields, please provide date for "decision made".
**2.2i**	Which date and time is recorded?	○ Decision to operate<br>○ First booked for theatre	
**2.3**	Consultant responsible for surgical care at the time the patient was booked for surgery (this may be different to the operating consultant)	(Local pick list of names with GMC number)	No longer collected in NELA from 1/12/19
**2.4**	Was there consultant surgeon input into the decision to operate?	○ Yes, consultant reviewed patient at time of decision*<br>○ Yes, following discussion with junior team member #<br>○ Decision made by junior team member **without** consultant input<br>○ Unknown	No longer collected in NELA from 1/12/19
**2.5**	NO LONGER REQUIRED	NO LONGER REQUIRED	
**2.6**	NO LONGER REQUIRED	NO LONGER REQUIRED	
**2.7**	Was an abdominal CT scan performed in the pre-operative period	○ Yes – reported by in-house consultant<br>○ Yes – reported by in-house	Combined with 2.7a from 1/12/19. Previous response options were yes/no/unknown

	as part of the diagnostic work-up?	registrar<br>○ Yes – reported by outsourced service<br>○ Yes but NOT reported<br>○ No CT performed<br>○ Unknown	
**2.7a**	If performed, how was this CT reported pre-operatively?	○ In-house consultant<br>○ In-house Registrar<br>○ Outsourced service<br>○ Not reported pre-operatively<br>○ Unknown	No longer collected in NELA from 1/12/19
**2.7b**	Was there a pre-operative discussion between the radiologist and the requesting team about the CT findings?	○ Yes<br>○ No<br>○ Unknown	No longer collected in NELA from 1/12/19
**2.7c**	Was there a discrepancy between the CT report and surgical findings that altered or delayed either the diagnosis or surgical management?	○ Yes<br>○ No<br>○ Unknown	No longer collected in NELA from 1/12/2020
**2.7d**	What was the Date and Time of CT Scan?	Date ____________(DD/MM/YYYY)<br>○ Date not known<br>Time_____________ (HH:MM)<br>○ Time not known	New question added in NELA 1/12/2020
**2.7e**	What was the Date and Time the CT Scan was reported electronically?	Date ____________(DD/MM/YYYY)<br>○ Date not known<br>Time_____________ (HH:MM)<br>○ Time not known	New question added in NELA 1/12/2020
**2.7f**	Was there an addendum added to the initial CT report which altered the patient pathway or the decision to proceed with surgery?	○ Yes - consultant addendum to SPR report<br>○ Yes - in house radiologist addendum to outsourced report<br>○ Yes - sub-specialist GI radiologist addendum non-GI consultant report<br>○ No<br>○ Unknown	New question added in NELA 1/12/2020
**2.8a**	Consultant Anaesthetist involvement in planning perioperative care	○ Yes – seen by consultant anaesthetist in person<br>○ Yes – discussion between consultant anaesthetist & other team member (of any specialty)<br>○ No consultant anaesthetist input before surgery<br>○ Unknown	No longer collected in NELA from 1/12/19

2.8b	Intensive care involvement in planning perioperative care	○ Yes – seen by consultant intensivist in person<br>○ Yes – discussion between consultant intensivist & other team member (of any specialty)<br>○ Seen by or discussion with junior ITU team member only<br>○ No intensive care input before surgery<br>○ Unknown	No longer collected in NELA from 1/12/19
2.9	NO LONGER REQUIRED	NO LONGER REQUIRED	
**2.10**	What was the date and time of the first dose of antibiotics following presentation to hospital?	○ In theatre, or<br>Date ____________(DD/MM/YYYY)<br>○ Date not known<br>Time_____________ (HH:MM)<br>○ Time not known<br>○ Not Administered	Only relevant for non-elective admissions
**2.11a**	Was sepsis, with a NEWS2 >=5 or >=3 in any one variable or another diagnosis requiring urgent antibiotics e.g. peritonitis / perforation, suspected on admission?	○ Yes<br>○ No<br>○ Unknown	
**2.11b**	Was sepsis, with a NEWS2 >=5 or >=3 in any one variable and/or another diagnosis requiring urgent antibiotics e.g. peritonitis / perforation, suspected at the time the decision for surgery was made?	○ Yes<br>○ No<br>○ Unknown	
**2.12**	On admission to hospital and using the Clinical Frailty Score what was the patients pre-admission frailty status assessed as being? (see help box for full pictorial explanation of each grading)	○ (1-3) - not frail<br>○ 4 - vulnerable<br>○ 5 - mildly frail<br>○ 6 - moderately frail<br>○ 7 - severely frail - completely dependent for personal care<br>○ 8 - very severely frail<br>○ 9 - Terminally ill<br>○ 0 - Not Recorded	Question wording changed from 1/12/2020 (previous version: “Using the Clinical Frailty Score (see help box), what was the patients pre-admission frailty status assessed as being?”)

3	**Pre-op Risk stratification**	**Format**	**Notes**
**3.1**	Prior to surgery, what was the risk of death for the patient that was entered into medical record?	○ Lower (<5%)<br>○ High (>=5%)<br>○ Not documented	For information, wording of relevant standard: "An assessment of mortality risk should be made explicit to the patient and recorded clearly on the consent form and in the medical record."<br><br>If both percentage predicted mortality AND risk category are documented, please select the highest risk option<br><br>Responses changed from 1/4/19. Previous versions: lower (<5%) / high (5-10%) / highest (>10%) / Not documented
**3.1a**	If documented, how was risk assessed?	○ Objective clinical score<br>○ Clinical judgement	New combined question from 1/12/19
**3.1b**	If patient assessed to be high risk, which consultants were involved immediately preoperatively in the assessment, decision making process and care of this patient? This may be either direct or indirect care. Please mark all that apply.	○ Consultant Surgeon<br>○ Consultant Anaesthetist<br>○ Consultant Intensivist<br>○ None	New combined question from 1/12/19
**3.2**	If documented, how was this assessment of risk made? (Please select all that apply)	○ Risk prediction tool (e.g. P-POSSUM)<br>○ Clinical Judgement<br>○ Surgical APGAR<br>○ Physiological criteria<br>Other e.g. hospital policy	No longer collected in NELA from 1/12/19
**3.3**	What was the **ASA** score?	○ 1: No systemic disease<br>○ 2: Mild systemic disease<br>○ 3: Severe systemic disease, not life-threatening<br>○ 4: Severe, life-threatening<br>○ 5: Moribund patient	
**3.4**	What was the most recent pre-operative value for serum Creatinine (micromol/l)	○ ____________<br>○ Not performed	Please enter values closest to time of booking for theatre
**3.5**	What was the most recent pre-operative value for blood lactate – may be arterial or venous (mmol/l)	○ ____________<br>○ Not performed	Please enter values closest to time of booking for theatre. Only one decimal point required.
**3.5i**	What was the highest CRP in the pre-	○ ____________<br>○ Not performed	No longer collected in NELA from 1/12/19

	operative period (mg/l)?		
**3.5ii**	What was the lowest albumin in the pre-operative period (g/l)?	o _____________<br>o Not performed	
	**P-POSSUM calculation**		No longer calculated within NELA from 1/4/19
	**NELA Risk calculation**		Added to NELA from 1/4/2019
	**For questions 3.6 to 3.22 please enter values closest to time of booking for theatre in order to calculate NELA Risk score. Answers should reflect chronic *and* acute pathophysiology**.<br>**in order to calculate**		
**3.6**	Serum Sodium concentration (mmol/l)		
**3.7**	Serum Potassium concentration (mmol/l)		
**3.8**	Serum Urea concentration (mmol/l)		
**3.9**	Serum Haemoglobin concentration (g/dl)		Units must be in g/l. If results are presented as g/dl in your institution, the value should be multiplied by 10 to convert to g/l.
**3.10**	Serum White cell count (x10^9 / l)		
**3.11**	Pulse rate(bpm)		
**3.12**	Systolic blood pressure (mmHg)		
**3.13**	Glasgow coma scale		
**3.14**	Select an option that best describes this patient's **ECG**	o No abnormalities<br>o AF rate 60-90<br>o AF rate >90/ any other abnormal rhythm/paced rhythm/ >5VE/min/ Q, ST or T wave abnormalities	If no investigation have been performed AND there is no clinical detail available, please select "no abnormality"
**3.15**	Select an option that best describes this patient's **cardiac signs** and chest xray appearance	o No failure<br>o Diuretic, digoxin, antianginal or antihypertensive therapy<br>o Peripheral oedema, warfarin Therapy or CXR: borderline cardiomegaly<br>o Raised jugular venous pressure or CXR: cardiomegaly	If CXR findings are worse than clinical findings, (or vice versa) please use worst score.<br>If no investigation have been performed AND there is no clinical detail available, please select "no abnormality"
**3.16**	Select an option that best describes this patient's **respiratory history** and chest xray appearance	o No dyspnoea<br>o Dyspnoea on exertion or CXR: mild COAD<br>o Dyspnoea limiting exertion to < 1 Flight or CXR: moderate COAD<br>o Dyspnoea at rest/rate >	If CXR findings are worse than clinical findings, (or vice versa) please use worst score.<br>If no investigation have been performed AND there is no clinical detail available, please select "no abnormality"

		30 at rest or CXR: fibrosis or consolidation	
3.16a	Patient was ventilated prior to emergency laparotomy	○ Yes<br>○ No	No longer collected within NELA
		Online web tool will automatically calculate Physiology severity score	
**3.17**	Select the **operative severity** of the intended surgical intervention (see help box for examples)	○ Major<br>○ Major+	**Major+:**<br>All colonic resections (excluding colostomy alone)<br>All gastrectomy (but not repair perforated or bleeding ulcer) Small bowel tumour resection<br>Re-operations for ongoing sepsis or bleeding<br>Laparostomy<br>Intestinal bypass<br>**Major**<br>All other procedures including: Stoma formation<br>Small bowel resection Division adhesions<br>Repair perforated or bleeding ulcer
**3.18**	Including this operation, how many operations has the patient had in the 30 day period prior to this procedure?	○ 1<br>○ 2<br>○ >2	Do not "unbundle" procedures. Examples of single procedure:<br>• Hartmann's procedure (this should not be "unbundled" as 2 procedures -sigmoid colectomy and end colostomy).<br>• Colonic resection with washout of a localised abscess would also be 1 procedure.<br>Examples of 2 procedures:<br>• Primary colonic anastomosis with a defunctioning ileostomy.<br>• Colonic resection and extensive division of adhesions.<br>• Colonic resection and small bowel repair.<br>Example of >2 procedures:<br>Hartmann's procedure **with** resection of small bowel **with**<br>insertion of tube gastrostomy
**3.19**	Based on your clinical experience of the intended surgery, please estimate the likely ***intra*operative blood loss** (ml)	○ <100<br>○ 101-500<br>○ 501-999<br>○ >=1000	Based on your clinical experience, please do your best to estimate the likely volume of intraoperative blood loss.
**3.20**	Please select a value that best describes the likely degree of	○ None<br>○ Serous fluid<br>○ Localised pus	Based on available radiological imaging and your clinical experience, please do your best to estimate the likely degree of peritoneal soiling.

	**peritoneal soiling**	○ Free bowel content, pus or blood	
**3.21**	What severity of malignancy is anticipated to be present?	○ None<br>○ Primary only<br>○ Nodal metastases<br>○ Distant metastases	Based on available radiological imaging and your clinical experience, please do your best to estimate the extent of intra- abdominal malignancy.
**3.22**	Please select urgency of surgical intervention (see help notes for additional information, including equivalent Possum categories)	○ 3. Expedited (>18 hours)<br>○ 2B. Urgent (6-18 hours)<br>○ 2A. Urgent (2-6 hours)<br>○ 1. Immediate (<2 hours)	Based on your clinical experience this should be the maximum time that a patient could reasonably wait for surgery. These classifications are based on NCEPOD and Surviving Sepsis. The equivalent POSSUM categories are also shown.<br><br>Examples:<br>**POSSUM: Emergency (resuscitation of > 2h possible)**<br>3. Expedited (>18 hours): No SIRS or sepsis e.g. developing large bowel obstruction<br>2B. Urgent (6-18 hours): Sepsis e.g. localised abscess or obstructed hernia<br>2A. Urgent (2-6 hours): Severe sepsis e.g. intestinal perforation<br><br>**POSSUM: Emergency (immediate surgery <2h needed)**<br>1. Immediate (<2 hours): Life threatening haemorrhage and septic shock e.g. profuse GI bleed or pan-intestinal ischaemia
		Online web tool will automatically calculate Operative severity score	
**3.23**	Pre-op P-POSSUM predicted mortality	Calculated______________	No longer calculated within NELA from 1/4/19
**3.24**	Pre-op POSSUM predicted morbidity	Calculated______________	No longer calculated within NELA from 1/4/19
**3.25**	Not all NELA investigations available	○	Wording changed 1/4/19 during switch from P-POSSUM to NELA risk score
**3.26**	Estimated mortality using NELA risk adjustment model	○ Calculated______________	Figure only provided if all data available

4	Intra-op	Format	Notes
**4.1**	Date and time of entry in to operating theatre/anaest	Date______________(DD/MM/YYYY)<br>Time_____________(HH:MM)<br>○ Time not known	Please enter the date/time at which the patient enters the anaesthetic room OR operating theatre (for patients anaesthetised in theatre),

	hetic room (not theatre suite)		whichever comes first.
**4.2**	Senior surgeon grade	○ Consultant<br>○ Post-CCT fellow<br>○ SAS grade<br>○ Research Fellow / Clinical Fellow<br>○ Specialty trainee / registrar<br>○ Core trainee / SHO<br>○ Other	This can include surgeon supervising in theatre but not necessarily scrubbed
**4.2a**	Consultant present/supervising: Name/GMC/specialty of operating or supervising consultant	(Please select consultant - Online)<br>______________________________	If consultant not present, enter name of supervising consultant
**4.3**	Senior anaesthetist present in theatre	○ Consultant<br>○ Post-CCT fellow<br>○ SAS grade<br>○ Research Fellow / Clinical Fellow<br>○ Specialty trainee / registrar<br>○ Core trainee / SHO<br>○ Other	
**4.3a**	Consultant present (or supervising): Name/GMC of anaesthetist	(Please select consultant - Online)<br>______________________________	If consultant not present, enter name of supervising consultant
**4.4**	How did you provide goal directed fluid therapy?	○ Patient recruited to FLO-ELA trial*<br>○ Not provided<br>○ Dynamic index e.g. Stroke volume, PPV, SVV<br>○ Static index e.g. CVP<br>○ Other, e.g. bioimpedance	PPV – pulse pressure variability<br>SVV – stroke volume variability<br>CVP – central venous pressure<br><br>*this response only available for NELA sites participating in FLO-ELA<br><br>4.4 removed from sites not participating in FLO-ELA from 1/12/19

**SECTION 4 (FLO-ELA supplementary questions)**		**Format**	**Notes**	**Help text**	**Visible for CONTROL patients**	**Visible for INTERVENTION**
**Heading**	**FLO-ELA Trial questions**					
**4.F.1**	FLO-ELA trial ID from	XXX-XXXXX			Y	Y

	randomisation system:					
**4.F.2**	To which treatment has the patient been randomised?	CONTROL group – usual care WITHOUT cardiac output monitoring, INTERVENTION group – cardiac output-guided haemodynamic therapy as per FLO-ELA algorithm			**Y**	**Y**
**4.F.3a**	Patient weight	........kg		Measured or best estimate	Y	Y
**4.F.3b**	Patient height	........cm	RANGE: 100 - 300	Measured or best estimate	**Y**	**Y**
**4.F.3c**	Ideal body weight	Calculated		For obese patients, consider administering maintenance fluid in ml/kg based on ideal body weight.	Y	Y

5	Procedure	Format	Notes
5.1	Is this the first surgical procedure of this admission, or a complication of previous surgery within the same admission?	○ Yes- First surgical procedure after admission<br>○ No - Surgery for complication of previous elective general surgical procedure within the same admission<br>○ No – Previous 'non-abdominal/non-general surgical' procedure within same admission (eg previous hip replacement)<br>○ Unknown	
**5.2**	What is the indication for surgery? (Please select all that apply)	○ Peritonitis<br>○ Perforation<br>○ Abdominal abscess<br>○ Anastomotic leak<br>○ Intestinal fistula<br>○ Phlegmon<br>○ Pneumoperitoneum<br>○ Necrosis<br>○ Sepsis<br>○ Small bowel obstruction<br>○ Large bowel obstruction	

		○ Volvulus<br>○ Internal hernia<br>○ Pseudo-obstruction<br>○ Intussusception<br>○ Incarcerated hernia<br>○ Obstructing incisional hernia<br>○ Haemorrhage<br>○ Hiatus Hernia/para-oesophageal hernia<br>○ Ischaemia<br>○ Colitis<br>○ Abdominal wound dehiscence<br>○ Abdominal compartment syndrome<br>○ Acidosis<br>○ Iatrogenic injury<br>○ Foreign body<br>○ Planned relook	
**5.3.a**	Main procedure	○ Peptic ulcer – suture or repair of perforation<br>○ Peptic ulcer – oversew of bleed<br>○ Gastrectomy: partial or total<br>○ Gastric surgery - other<br>○ Small bowel resection<br>○ Resection of Meckel's diverticulum<br>○ Colectomy: left (including sigmoid colectomy and anterior resection)<br>○ Colectomy: right (including ileocaecal resection)<br>○ Colectomy: subtotal or panproctocolectomy<br>○ Hartmann's procedure<br>○ Colorectal resection - other<br>○ Abdominal wall closure following dehiscence<br>○ Abdominal wall reconstruction<br>○ Adhesiolysis<br>○ Reduction of volvulus<br>○ Enterotomy<br>○ Stricturoplasty<br>○ Drainage of abscess/collection<br>○ Evacuation of haematoma<br>○ Debridement<br>○ Exploratory/relook laparotomy only<br>○ Haemostasis<br>○ Intestinal bypass<br>○ Laparostomy formation<br>○ Repair of intestinal perforation<br>○ Repair or revision of anastomosis<br>○ Repair of intestinal fistula<br>○ Resection of other intra-abdominal tumour(s)	Please note that, in accordance with NELA inclusion criteria, primary and additional procedure options vary<br><br>Please see inclusion/exclusion criteria under the "support" tab on this data collection website. They can also be downloaded from http://www.nela.org.uk/NELA_Docs
**5.3.b**	Second procedure (at same laparotomy)		
5.3.c	Third procedure (at same laparotomy)		

		○ Defunctioning stoma via midline laparotomy<br>○ Revision of stoma via midline laparotomy<br>○ Large incisional hernia repair with bowel resection<br>○ Large incisional hernia repair with division of adhesions<br>○ Washout only<br>○ Removal of foreign body<br>○ Not amenable to surgery<br>○ Removal of gastric band<br>○ Repair of para-oesphageal hernia<br>○ Splenectomy	
**5.3e**	Was a stoma formed (by any means)?	○ Yes<br>○ No	Added to NELA from 1/12/2019
**5.4**	Procedure approach	○ Open<br>○ Laparoscopic<br>○ Laparoscopic assisted<br>○ Laparoscopic converted to open	
**5.5**	Operative findings:<br>(Please select all that apply)<br>If unsure whether this patient is eligible for NELA please refer to help box	○ Abscess<br>○ Anastomotic leak<br>○ Perforation – peptic ulcer<br>○ Perforation – small bowel/colonic<br>○ Diverticulitis<br>○ Intestinal fistula<br>○ Adhesions<br>○ Incarcerated hernia<br>○ Volvulus<br>○ Internal hernia<br>○ Intussusception<br>○ Stricture<br>○ Pseudo-obstruction<br>○ Gallstone ileus<br>○ Meckel's diverticulum<br>○ Malignancy – localised<br>○ Malignancy – disseminated<br>○ Colorectal cancer<br>○ Gastric cancer<br>○ Haemorrhage – peptic ulcer<br>○ Haemorrhage - intestinal<br>○ Haemorrhage – postoperative<br>○ Ulcerative colitis<br>○ Other colitis<br>○ Crohn's disease<br>○ Abdominal compartment syndrome<br>○ Intestinal ischaemia<br>○ Necrotising fasciitis<br>○ Foreign body<br>○ Stoma complications<br>○ Abdominal wound dehiscence	Operative findings are intended to be best guess. There may be instances where the operative findings are such that, had these findings been known prior to surgery, the patient would not have been included in the audit. However since they have now had a laparotomy, they are still included. This is why there appear to be some findings/procedures that are under the exclusion criteria.

		○ Normal intra-abdominal findings	
**5.6**	Please describe the peritoneal contamination present (select all that apply)	○ None or reactive serous fluid only<br>○ Free gas from perforation +/- minimal contamination<br>○ Pus<br>○ Bile<br>○ Gastro-duodenal contents<br>○ Small bowel contents<br>○ Faeculent fluid<br>○ Faeces<br>○ Blood/haematoma	
**5.7**	Please indicate if the contamination was;	○ Localised to a single quadrant of the abdomen<br>○ More extensive / generalised	

**6**	**End of Surgery**	**Format**	**Notes**
**6.1**	At the end of surgery, what risk of death was the patient documented as having?	○ Lower (<5%)<br>○ High (>=5%)<br>○ Not documented	Responses changed from 1/4/19. Previous versions: lower (<5%) / high (5-10%) / highest (>10%) / Not documented
**6.1a**	If documented, how was risk assessed?	○ Objective clinical score<br>○ Clinical judgement	New combined question from 1/12/19
**6.2**	If documented, how was this assessment of risk made? (Please select all that apply)	○Risk prediction tool (e.g. P-POSSUM)<br>○Clinical Judgement<br>○Surgical APGAR<br>○Physiological criteria<br>○Other e.g. hospital policy	No longer collected in NELA from 1/12/19
**6.3**	Blood lactate – may be arterial or venous (mmol/l)	______________<br>○ Not performed	Or within 30 minutes of the end of surgery.
	**Post-operative NELA Risk calculation**<br>*Q 6.4-6.14 no longer included from Year 4 specification*		P-POSSUM changed to NELA risk score from 1/4/2019
	Physiology severity score:	(Automatically calculated)	
**6.15**	What was the operative severity? (see help box for examples)	○ Major<br>○ Major+	**Major+:**<br>All colonic resections (excluding colostomy alone)<br>All gastrectomy (but not repair perforated or bleeding ulcer)<br>Small bowel tumour resection<br>Re-operations for ongoing sepsis or bleeding Laparostomy<br>Intestinal bypass

			**Major**<br>All other procedures including:<br>Stoma formation<br>Small bowel resection Division adhesions<br>Repair perforated or bleeding ulcer
**6.16**	Including this operation, how many operations has the patient had in the 30 day period prior to this procedure?	○ 1<br>○ 2<br>○ >2	Do not "unbundle" procedures.<br>Examples of single procedure:<br>• Hartmann's procedure (this should not be "unbundled" as 2 procedures -sigmoid colectomy and end colostomy).<br>• Colonic resection with washout of a localised abscess would also be 1 procedure.<br><br>Examples of 2 procedures:<br>• Primary colonic anastomosis with a defunctioning ileostomy.<br>• Colonic resection and extensive division of adhesions.<br>• Colonic resection and small bowel repair.<br><br>Example of >2 procedures:<br>• Hartmann's procedure **with** resection of small bowel **with** insertion of tube gastrostomy
**6.17**	Please select this patient's measured intraoperative blood loss (ml)	○ <100<br>○ 101-500<br>○ 501-1000<br>○ >1000	If measured blood loss is unavailable, please estimate
6.17a	If the patient's blood loss was greater than 500mls, was Tranexamic Acid given?	○ Yes<br>○ No	**Added to NELA from 1/12/2019**<br>**Not shared with FLO-ELA**
**6.18**	Please select the option that best describes this patient's degree of peritoneal soiling	○ None<br>○ Serious fluid<br>○ Local pus<br>○ Free bowel content, pus or blood	
**6.19**	What was the level of malignancy based on surgical findings	○ None<br>○ Primary only<br>○ Nodal metastases<br>○ Distant metastases	
**6.20**	What is the NCEPOD urgency?	○ 3. Expedited (>18 hours)<br>○ 2B. Urgent (6-18 hours)	Based on your clinical experience

	(see help notes for additional information, including equivalent Possum categories)	○ 2A. Urgent (2-6 hours)<br>○ 1. Immediate (<2 hours)	this should be the maximum time that a patient could reasonably wait for surgery. These classifications are based on NCEPOD and Surviving Sepsis. The equivalent POSSUM categories are also shown.<br><br>Examples:<br>**POSSUM: Emergency (resuscitation of > 2h possible)**<br>3. Expedited (>18 hours): No SIRS or sepsis e.g. developing large bowel obstruction<br>2B. Urgent (6-18 hours): Sepsis e.g. localised abscess or obstructed hernia<br>2A. Urgent (2-6 hours): Severe sepsis e.g. intestinal perforation<br><br>**POSSUM: Emergency (immediate surgery <2h needed)**<br>1. Immediate (<2 hours): Life threatening haemorrhage and septic shock e.g. profuse GI bleed or pan-intestinal ischaemia
	Online web tool will automatically calculate Operative severity score		
6.21	Post-op P-POSSUM predicted **mortality**:	Calculated __________	No longer calculated within NELA from 1/4/2019
6.22	Post-op POSSUM predicted **morbidity**:	Calculated __________	No longer calculated within NELA from 1/4/2019
**6.23**	Not all investigations available for calculation of NELA Risk	○	P-POSSUM changed to NELA from 1/4/2019
**6.24**	Where did the patient go for continued post-operative care following surgery?	○ Ward<br>○ Critical Care (includes Level 2 HDU or Level 3 ICU)<br>○ Extended recovery area within theatres (e.g. PACU or OIR)<br>○ Enhanced care area on a normal ward<br>○ Died prior to discharge from theatre complex	"Other enhanced care area (e.g. PACU)" expanded from 1/12/2019 to include Extended recovery area within theatres (e.g. PACU or OIR) / Enhanced care area on a normal ward
**6.24a**	At the end of surgery, was the decision made to place the patient on an end of life pathway?	○ Yes<br>○ No	This is intended to identify those patients whose pathology, at the time of surgery, was such that only supportive treatment was warranted.
**6.26**	Estimated mortality using NELA risk adjustment model	Calculated __________	

	(Figure only provided if all data available)		

SECTION 6	End of Surgery (FLO-ELA Trial questions)	Format	Notes	Help text	Visible for CONTROL patients	Visible for INTERVENTION patients
**Heading and subtitle**	**DURING surgery.** These questions relate to the intraoperative period. This is from the start of general anaesthesia to the end of surgery.					
**6.F.1**	Date and time of the start of general anaesthesia	Date____________(DD/MM/YYYY)<br>Time_____________ (HH:MM)		If the patient was fully sedated and intubated prior to arrival in the theatre suite for surgery, please indicate this. The start of the intraoperative period will be taken as the date/time when the patient arrived in the anaesthetic room/theatre (question 4.1) so please ensure this question is fully completed.	Y	Y
**6.F.1a**		○ Not applicable: patient fully sedated and intubated prior to arrival in the theatre suite				
**6.F.2**	Which cardiac output monitor was used?	○ Deltex Oesophageal Doppler<br>○ Edwards EV1000/FloTrac<br>○ LiDCO Rapid<br>○ LiDCO Plus<br>○ Not used *(this is a protocol deviation)*			N	Y
**6.F.3**	Date and time of start of	Date____________(DD/MM/YYYY)			N	Y

	cardiac output-guided haemodynamic therapy:	Time______________ (HH:MM)				
**6.F.4**	Date and time of the end of surgery	Date____________(DD/MM/YYYY)<br><br>Time______________ (HH:MM)			Y	Y
Subtitle:	**Maintenance fluid during surgery**				Y	Y
**6.F.5**	Fluid type	○ 5% dextrose<br>○ 4% dextrose with 0.18% NaCl (+/- KCl)<br>○ 5% dextrose with 0.45% NaCl (+/- KCl)<br>○ "Balanced" crystalloid<br>○ 0.9% sodium chloride<br>○ Other		If the type of maintenance fluid was changed during the intervention period, please select the fluid type that was given in the greatest quantity.	Y	Y
**6.F.5a**	Total maintenance fluid volume given during surgery	......................ml	RANGE: 0 - 20000			
Subtitle:	**Fluid boluses during surgery**				Y	N
Subtitle:	**Cardiac output-guided fluid boluses during surgery**				N	Y
6.F.6	How many fluid boluses were given in accordance with the FLO-ELA intervention algorithm during surgery?	…….	Number format, RANGE 0-100		N	Y
**6.F.7**	Please state the volume of each of the following fluids given as boluses during surgery:				Y	Y
**6.F.7 a**	"Balanced" crystalloid	……………ml	RANGE: 0 - 20000	"Balanced" crystalloids include Hartmann's solution (compound	Y	Y

				sodium lactate, Ringer's lactate), Plasmalyte 147.		
**6.F.7 b**	0.9% sodium chloride	....................ml	RANGE: 0 – 20000		Y	Y
**6.F.7 c**	Gelatin-based colloid	..............ml	RANGE: 0 - 20000		Y	Y
**6.F.7 d**	Albumin	.....................ml	RANGE: 0 – 20000		Y	Y
**6.F.7 e**	Red blood cells	...............ml	RANGE: 0 - 20000	If exact volume is not known, please calculate from the number of units given and the average adult red cell unit volume (280ml)	Y	Y
**6.F.7 f**	Other blood products	.....................ml	RANGE: 0 – 20000		Y	Y
**6.F.8**	Select any of the following drugs that were used (tick all that apply):	○ Vasopressors by bolus<br>○ Vasopressors by infusion<br>○ Inotropes by bolus<br>○ Inotropes by infusion<br>○ None of the above		For trial purposes these drugs are defined as follows:<br><br>*Vasopressors:* Metaraminol, phenylephrine, noradrenaline (norepinephrine), vasopressin<br>*Inotropes:* Ephedrine, dobutamine, dopexamine, dopamine, adrenaline (epinephrine), levosimendan	Y	Y
**6.F.9**	Was a cardiac output monitor	○ No<br>○ Yes		This includes any form of cardiac	Y	N

	used in a control group patient? *This is a protocol deviation*			output monitoring able to display stroke volume, stroke volume variation, or systolic/pulse pressure variation.		
**6.F.9 a**	**IF YES:** Date and time that cardiac output monitoring was started:	Date____________(DD/MM/YYYY)<br>Time_____________ (HH:MM)			Y	N
**6.F.9b**	Indication for cardiac output monitoring:	○ Patient deterioration<br>○ Other			Y	N
**6.F.9.c**	Please specify	....				

	**SECTION 7: Post-op FLO-ELA trial questions.**<br>**Relating to the period DURING THE SIX HOURS AFTER surgery:**				**Visible for CONTROL patients**	**Visible for INTERVENTION patients**
**7.F.1**	Which cardiac output monitor was used?	○ Deltex Oesophageal Doppler<br>○ Edwards EV1000/FloTrac<br>○ LiDCO Rapid<br>○ LiDCO Plus<br>○ Not used			N	Y
Subtitle:	**Maintenance fluid after surgery**				Y	Y
**7.F.2**	Fluid type	○ 5% dextrose<br>○ 4% dextrose with 0.18% NaCl (+/- KCl)<br>○ 5% dextrose with 0.45% NaCl (+/- KCl)<br>○ "Balanced" crystalloid<br>○ 0.9% sodium chloride<br>○ Other		If the type of maintenance fluid was changed during the intervention period, please select the fluid type that was given in the greatest quantity.	Y	Y
**7.F.2 a**	Total maintenance fluid volume given after	......................ml	RANGE:<br>(0-20000)		Y	Y

	surgery					
Subtitle:	**Fluid boluses after surgery**				Y	N
Subtitle:	**Cardiac output-guided fluid boluses after surgery**				N	Y
7.F.3	How many fluid boluses were given in accordance with the FLO-ELA intervention algorithm in the six hours after surgery?	……	Number format<br>RANGE: 0-100		N	Y
**7.F.4**	Please state the volume of each of the following fluids given as boluses after surgery:				Y	Y
**7.F.4 a**	"Balanced" crystalloid	……………ml	RANGE: 0 - 20000	"Balanced" crystalloids include Hartmann's solution (compound sodium lactate, Ringer's lactate), Plasmalyte 148.	Y	Y
**7.F.4 b**	0.9% sodium chloride	…………………ml	RANGE: 0 – 20000		Y	Y
**7.F.4 c**	Gelatin-based colloid	………….ml	RANGE: 0 - 20000		Y	Y
**7.F.4 d**	Albumin	…………………ml	RANGE: 0 – 20000		Y	Y
**7.F.4 e**	Red blood cells	……………ml	RANGE: 0 - 20000	If exact volume is not known, please calculate from the number of units given and the average adult red cell unit volume (280ml)	Y	Y
**7.F.4 f**	Other blood products	…………………ml	RANGE: 0 – 20000		Y	Y

**7.F.5**	Select any of the following drugs that were used (tick all that apply):	○ Vasopressors by bolus<br>○ Vasopressors by infusion<br>○ Inotropes by bolus<br>○ Inotropes by infusion<br>○ None of the above		For trial purposes these drugs are defined as follows:<br>*Vasopressors:*<br>Metaraminol, phenylephrine, noradrenaline (norepinephrine), vasopressin<br>*Inotropes:*<br>Ephedrine, dobutamine, dopexamine, dopamine, adrenaline (epinephrine), levosimendan	Y	Y
**7.F.6**	Was a cardiac output monitor used in a control group patient? *This is a protocol deviation*	○ No<br>○ Yes		This includes any form of cardiac output monitoring able to display stroke volume, stroke volume variation, or systolic/pulse pressure variation.	Y	N
**7.F.6 a**	**IF YES:** Date and time that cardiac output monitoring was started:	Date____________(DD/MM/YYYY)<br>Time_____________ (HH:MM)			Y	N
**7.F.6 b**	Indication for cardiac output monitoring:	○ Patient deterioration<br>○ Other			Y	N
**7.F.6 c**	Please specify	....	RANGE: (max 100chars)		Y	N
**7.F.7**	Date and time of the end of cardiac output-guided haemodynamic therapy:	Date____________(DD/MM/YYYY)<br>Time_____________ (HH:MM)		The trial intervention should continue for six hours after surgery where possible. In cases where this has not been possible please give the reason below.	N	Y

**7.F.8a**	Was cardiac output-guided therapy stopped before six hours had elapsed after the end of surgery?	○ No<br>○ Yes			N	Y
**7.F.8b**	Please state reason:	○ Patient transferred to Level 1 care<br>○ Unable to deliver ongoing intervention in Post-Anaesthetic Care Unit<br>○ Other (state):	"Other" text max 200 characters			
**7.F.9**	**For completion by the local FLO-ELA research team when patient is discharged from hospital:** Please confirm patient consent (select one choice)	○ Prospective patient consent<br>○ Prospective consultee or emergency agreement followed by retrospective patient consent<br>○ Prospective emergency agreement followed by retrospective consultee agreement (patient unable to provide consent)<br>○ Prospective consultee or emergency agreement – unable to obtain retrospective consent before discharge		Please see protocol and standard operating procedures for definitions.	Y	Y

**7**	**Post-op**	**Format**	**Notes**
**7.1**	Total length of post-operative critical care stay (rounded up to whole days)<br>*Includes both ICU and HDU stay -see help box for additional information. Do not include LOS in PACU/other enhanced recovery area*	____________<br>Number required	Each day, or part day, counts as 1 day. Hence:<br><br>a. Admitted and discharged on same day = 1 day<br>b. Admitted on Monday, discharged on Tues = 2 days<br>c. Admitted on Monday, discharged on Wed = 3 days.<br>Values should reflect actual discharge, rather than when medically fit for discharge.
7.2	NO LONGER REQUIRED		
**7.3**	For frail (CFS≥5) patients aged 65 or older, was the patient assessed by a member of the geriatrician-led multidisciplinary team during any part of the perioperative period?	○ Yes<br>○ No<br>○ Unknown	Can include physician or nurse specialist<br><br>Question wording changed from 1/12/2019 (previous version:

			"Was the patient assessed by a specialist from Elderly Medicine in the post-operative period?<br><br>Question wording changed from 1/12/2020 (previous version: "For patients aged 65 or older, was the patient assessed by a consultant geriatrician during any part of the perioperative period")
**7.4**	Within this admission, did the patient have an **unplanned or planned** return to theatre in the post-operative period following their initial emergency laparotomy?	o Yes; unplanned return<br>o Yes; planned return<br>o Yes; unplanned AND planned return<br>o No<br>o Unknown	Question combined from 1/12/2019. Previous question only covered unplanned returns (responses yes/no)
**7.4a**	What was the main indication for the **unplanned** return to theatre	o Anastomotic leak<br>o Abscess<br>o Bleeding or Haematoma<br>o Decompression of abdominal compartment syndrome<br>o Bowel obstruction<br>o Abdominal wall dehiscence<br>o Accidental damage to bowel or other organ<br>o Stoma viability or retraction<br>o Other<br>o Unknown	"unplanned" added from 1/12/2019 to combine 7.4 sub-questions.
7.4b	NO LONGER REQUIRED	o	
**7.5**	Did the patient have an unplanned move **from the ward** to a higher level of care within 7 days of surgery? (do not include moves from HDU to ITU, or escalation from other enhanced area/PACU)	o Yes<br>o No<br>o Unknown	This refers to within 7 days of their emergency laparotomy, not any prior surgery.
**7.6**	NO LONGER REQUIRED	NO LONGER REQUIRED	
**7.7**	Status at discharge	o Dead<br>o Alive<br>o Still in hospital at 60 days	'Still in hospital at 60 days' option to be used when approaching an audit deadline by which all incomplete cases need to be locked
**7.8**	Date discharged from hospital	______________<br>(DD/MM/YYYY)<br>Date required	Date of discharge, NOT date fit for discharge.<br>**Only shared with FLO-ELA for those discharged alive,** to avoid generating date of death as a direct patient identifier
**7.9**	Discharge destination	o Own home/sheltered housing<br>o Residential care<br>o Nursing care<br>o Unknown	No longer collected in NELA from 1/12/2019

	**COVID-19 Questions**		New Questions added in 2020
**7.10**	Please indicate the patient's SARS-CoV-2/COVID-19 infection status	o Covid positive – confirmed pre-operatively<br>o Covid positive – confirmed post-operatively<br>o Covid negative throughout in-patient stay	
**7.11**	NO LONGER REQUIRED	NO LONGER REQUIRED	
**7.12**	NO LONGER REQUIRED	NO LONGER REQUIRED	

Appendix 3 - Level of care after surgery

The level of care should be defined according to the care the patient received rather than the location. For example, a patient receiving level 2 care in a level 3 area should be recorded as receiving level 2 care.

1. Critical care level 3: includes advanced organ support e.g. invasive ventilation, renal replacement therapy.
2. Critical care level 2: may include advanced cardiorespiratory monitoring (e.g. invasive arterial / central venous monitoring) and basic organ support (e.g. non-invasive ventilation, inotropic/vasoactive drug administration).
3. Post-anaesthetic care unit: care within a designated area for the patients in the immediate recovery from anaesthesia. May deliver care at levels 1 to 3.

Surgical ward (level 0/1): normal ward care without level 2 or 3 capabilities.

Appendix 4 – Typical complications of emergency laparotomy

The following are recognised complications of emergency laparotomy surgery, which may have clinical severity up to and including disability and death.

Acute kidney injury
Acute Respiratory Distress Syndrome (ARDS)
Anaphylaxis
Anastamotic breakdown
Bowel infarction
Cardiac arrhythmia
Cardiac arrest
Cardiogenic pulmonary oedema
Deep vein thrombosis
Delirium or acute psychosis
Electrolyte imbalance
Gastrointestinal or other postoperative bleed
Infection, source uncertain
Laboratory confirmed bloodstream infection
Multi-organ dysfunction syndrome
Myocardial infarction
Myocardial injury after non-cardiac surgery
Pneumonia
Paralytic ileus
Perforated viscus
Postoperative haemorrhage
Pulmonary embolism
Stroke
Surgical site infection (superficial, deep or organ/space)
Urinary tract infection

Appendix 5 – Protocol version history

Protocol:		Amendments:		
**Version no.**	**Date**	**Amendment no.**	**Protocol section (no./title)**	**Summary of main changes from previous version**
1.0	26/01/2017	N/A	N/A	N/A
2.0	13/09/2019	1	Summary<br><br>5.6 Health economic endpoints, 7.7.4 Outcomes data, 7.12 schedule of assessments, 8.3 Health economic analysis<br><br>6.2 Inclusion criteria<br><br>All references to NHS number<br><br>All references to NELA data collection tool	Revised to reflect addition of sites in Scotland/NI. Proposed end date and study duration revised.<br><br>Revised to add one-year time-point to health economic analysis, further revisions to clarify health economic analysis plan.<br><br>Full list of eligible procedures moved to Appendix 1. Definition of "expedited surgery" corrected. Added interpretation of eligible procedures for Scotland/NI<br><br>Now incorporates NHS (England & Wales), Community Health Index (CHI - Scotland) /Health and Care (H&C – Northern Ireland) numbers<br><br>Updated to reflect alternative arrangement to be used in Scotland/NI<br><br>Revised approach to recruitment of patients not capable of giving consent. Added procedures for recruiting patients with incapacity in Scotland.<br><br>Revised for clarity

			7.1 Recruitment and screening<br><br>7.4 Trial treatment<br><br>7.6 Blinding and procedures to minimize bias<br><br>7.7 Data collection<br><br>Appendix 1<br><br>Appendix 2	Added detail confirming no access to unblinded trial data until final database lock.<br><br>Added detail on data controllers for trial data sources, and alternative arrangements for data collection in Scotland and NI.<br><br>Updated in line with changes to NELA inclusion/exclusion criteria<br><br>Updated in line with changes to NELA dataset
3.0	22/09/2021	2	Title page<br><br>3 Summary, 4 Introduction, 5 Trial Objectives, 6.4.3 Results from internal pilot, 7.6 Blinding and procedures to minimise bias, 8 Statistical considerations.<br><br>Appendix 2	Updated NIHR logo<br><br>Updated with modified primary/secondary outcomes and revised sample size<br><br>Updated in line with changes to NELA dataset

4.0	27/04/2022	3	3 Summary, 5.9 Estimand framework, 7.10 Withdrawals, 8.2 Statistical analysis	Updated (5.9 added) in line with main trial analyses being carried out under the estimand framework, with patients not receiving surgery excluded from the analyses. Additional exploratory analyses planned to look at the potential impact of Covid-19 on treatment effect, NELA-defined standards of care and risk profile of trial participants.
5.0	14/11/2024	4	3. Summary, 5.7. Assessment of Primary and Secondary Outcomes, 8.2 Statistical analysis<br>15. Finance and Funding	Updated NIHR logo<br>Updated project duration and end date<br>Amended reference to National Institute for Health Research, and replaced to National Institute for Health and Care Research<br><br>Updated data captured from HES, NSSISD, NI Healthcare Trusts and PEDW data to include the duration of the hospital stay during the index hospital admission as well as during any hospital readmissions.<br><br>Included the following funding statement: This study/ project is funded by the NIHR Health Technology Assessment Programme (15/80/54). The views expressed are those of the author (s) and not necessarily those of the NIHR or the Department of Health and Social Care.

				Changed model of data analysis from mixed effect negative binomial to linear regression (mixed effect also)
